# Human neutrophil IL1β directs intestinal epithelial cell extrusion during *Salmonella* infection

**DOI:** 10.1371/journal.ppat.1010855

**Published:** 2022-10-03

**Authors:** Anna-Lisa E. Lawrence, Ryan P. Berger, David R. Hill, Sha Huang, Veda K. Yadagiri, Brooke Bons, Courtney Fields, Gautam J. Sule, Jason S. Knight, Christiane E. Wobus, Jason R. Spence, Vincent B. Young, Mary X. O’Riordan, Basel H. Abuaita

**Affiliations:** 1 Department of Microbiology and Immunology, University of Michigan Medical School, Ann Arbor, Michigan, United States of America; 2 Department of Internal Medicine, University of Michigan Medical School, Ann Arbor, Michigan, United States of America; 3 Department of Cell and Developmental Biology, University of Michigan Medical School, Ann Arbor, Michigan, United States of America; University of California Davis School of Medicine, UNITED STATES

## Abstract

Infection of the human gut by *Salmonella enterica* Typhimurium (STM) results in a localized inflammatory disease that is not mimicked in murine infections. To determine mechanisms by which neutrophils, as early responders to bacterial challenge, direct inflammatory programming of human intestinal epithelium, we established a multi-component human intestinal organoid (HIO) model of STM infection. HIOs were micro-injected with STM and seeded with primary human polymorphonuclear leukocytes (PMN-HIOs). PMNs did not significantly alter luminal colonization of *Salmonella*, but their presence reduced intraepithelial bacterial burden. Adding PMNs to infected HIOs resulted in substantial accumulation of shed TUNEL^+^ epithelial cells that was driven by PMN Caspase-1 activity. Inhibition of Caspases-1, -3 or -4 abrogated epithelial cell death and extrusion in the infected PMN-HIOs but only Caspase-1 inhibition significantly increased bacterial burden in the PMN-HIO epithelium. Thus, PMNs promote cell death in human intestinal epithelial cells through multiple caspases as a protective response to infection. IL-1β was necessary and sufficient to induce cell shedding in the infected HIOs. These data support a critical innate immune function for human neutrophils in amplifying cell death and extrusion of human epithelial cells from the *Salmonella*-infected intestinal monolayer.

## Author summary

Neutrophils are early responders to *Salmonella* intestinal infection, but how they influence infection progression and outcome is unknown. Here we use a co-culture model of human intestinal organoids and human primary neutrophils to study the contribution of human neutrophils to *Salmonella* infection of the intestinal epithelium. We found that neutrophils markedly enhanced epithelial defenses, including enhancing cell extrusion to reduce intraepithelial burden of *Salmonella* and close association with the epithelium. These findings reveal an early role for neutrophils in the gut in shaping the gut environment to control epithelial infection.

## Introduction

*Salmonella enterica* is one of the most common causes of foodborne disease, responsible for an estimated 1.35 million infections in the United States each year [1]. *S*. *enterica* serovar Typhimurium (STM), one of the most prevalent *S*. *enterica* serovars, infects via the fecal-oral route and stimulates robust inflammation in humans, leading to gastroenteritis and diarrheal disease [2]. In contrast, oral infection of C57Bl/6 mice with *S*. Typhimurium leads to a systemic infection that more closely resembles infection by the human-specific pathogen. *S*. Typhi, and is therefore often used as a model for typhoid fever. In order to recapitulate the human inflammatory intestinal disease caused by *S*. Typhimurium in a mouse model, pretreatment of animals with streptomycin or dextran sodium sulfate is commonly used to generate an inflammatory environment that promotes STM luminal replication. The need for exogenous inflammatory insult to establish STM-associated gastroenteritis in the mouse suggests that the corresponding human innate immune response to STM deploys a robust early inflammatory signal that shapes the initial response of the intestinal epithelium.

Prior studies have elucidated an important cell-intrinsic role of the inflammasome in intestinal epithelium [3]. Sellin et al. and Rauch et al. showed that murine intestinal epithelial cells (IEC) respond to STM infection by activating NAIP5 and NLRC4 inflammasomes, leading to epithelial cell extrusion [4,5]. Of note, STM-infected human intestinal epithelial cells also activate the inflammasome but rely on Caspase-4 rather than NAIP5, NLRC4 or NLRP3 or Caspase-1, highlighting key differences between murine and human epithelial innate immune responses [6–9]. While these epithelial responses favor the host, only about 15–30% of infected cells are extruded, leaving a significant proportion of infected cells in the epithelium [6,8]. The contribution of other cell types, such as innate immune cells, in regulating activation of programmed cell death pathways and shedding of intestinal epithelial cells has not been fully explored in *Salmonella* infection. Innate immune cells such as polymorphonuclear leukocytes (PMNs) can induce cell death of epithelial cells under inflammatory conditions [10,11], suggesting that immune cells recruited to *Salmonella*-infected intestines may aid in enhancing epithelial defenses during infection.

PMNs can defend against bacterial infections through direct and indirect mechanisms. Antimicrobial effectors like degradative proteases and ion chelators, production of reactive oxygen species and formation of sticky antimicrobial neutrophil extracellular traps (NETs) in many cases can also serve as signaling mediators [12]. However, it is becoming appreciated that PMNs serve critical inflammatory functions, e.g., changing the microenvironment via molecular oxygen depletion, regulating nutrient availability, and through production of inflammatory mediators [13,14]. Notably, Gopinath et al, found that neutrophilia induced a super-shedder phenotype in a mouse infection model of *Salmonella* [15], but how the interaction between epithelial cells and PMNs affects the outcome of bacterial infections is still poorly defined. To address this gap in knowledge, we generated a co-culture model of primary human PMNs, specifically neutrophils, with human intestinal organoids (HIOs) termed PMN-HIOs to study the contribution of PMNs during infection with *Salmonella enterica* serovar Typhimurium (STM).

Using this PMN-HIO co-culture model, we evaluated how PMNs modulate intestinal epithelial host defenses during infection, compared to infected HIOs alone. We show here that the presence of PMNs elevates the overall inflammatory tone of the epithelium and markedly promotes cell death and extrusion of epithelial cells, thereby reducing *Salmonella* intraepithelial burden.

## Results

### Human PMNs transmigrate into the HIO lumen during infection and reduce *Salmonella* intraepithelial burden

**Fig 1:**
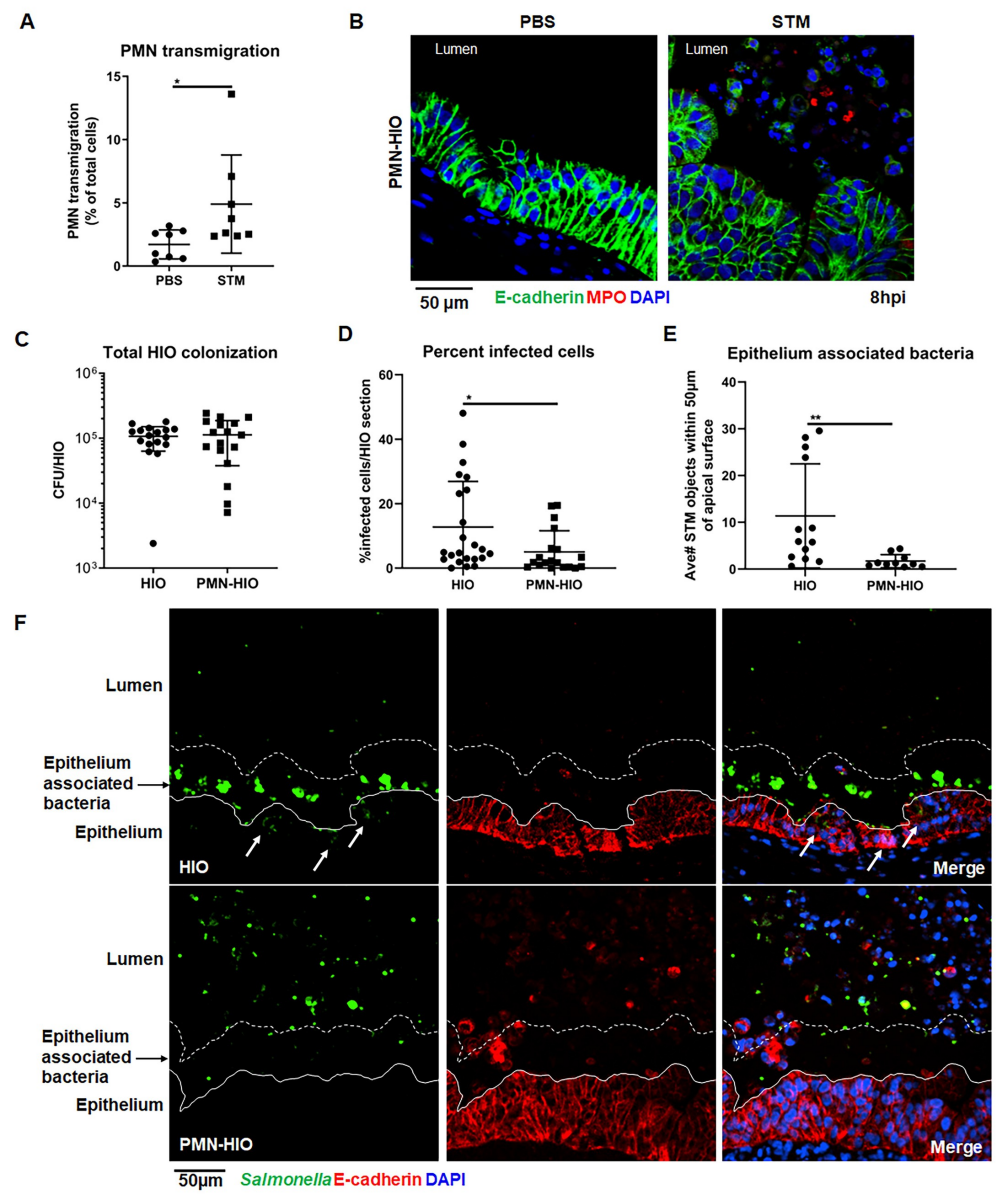
PMNs migrate into the lumen during infection and reduce both the number of infected epithelial cells and the association of bacteria with the epithelial surface. A. Transmigration of PMNs into the lumen of HIOs was quantified using flow cytometry. HIOs were microinjected with STM or PBS and co-cultured with CFSE-labeled PMNs for 8h. PMNs-HIOs were washed to remove any unassociated PMNs, dissociated into a single cell suspension and subjected to flow cytometry. Percentage of PMNs relative to total cells acquired per PMN-HIO was determined by FlowJo software. B. Immunofluorescent staining of HIOs microinjected with PBS or STM and co-cultured with PMNs. E-cadherin (green) marks the epithelial lining, MPO (red) is specific to PMNs and DNA is stained with DAPI. C. Total bacterial burden per HIO or PMN-HIO was enumerated at 8hpi. Individual HIOs were removed from Matrigel, washed with PBS and homogenized in PBS. Serial dilutions were plated on LB agar to enumerate bacterial burden. D. Quantitation of percent infected cells/HIO or PMN-HIO based on 3 fields per view per HIO. E. Quantitation of epithelium associated bacteria. Number of bacteria within 50μm of the apical epithelial surface based on E-cadherin staining were counted and normalized per 100μm distance. F. Representative immunofluorescent staining of HIOs and PMN-HIOs infected with STM. *Salmonella* is stained in green, E-cadherin to mark epithelial cells is shown in red, and DNA stained with DAPI in blue. White arrows point to infected cells. Graphs show the mean and SD of n≥ 10 HIOs represented by dots from at least two independent experiments. Outliers were removed using the ROUT method with Q = 0.1%. Unless otherwise stated, significance was determined by Mann-Whitney test with *p<0.05, **p<0.01.

PMNs are recruited to and transmigrate across intestinal epithelial layers during early stages of inflammation [16,17]; therefore we asked whether PMNs would transmigrate into the HIO lumen during infection. *Salmonella enterica* Typhimurium (STM; 10^5^ CFU) was microinjected into the HIO lumen and cultured for 8h with PMNs isolated from healthy human volunteers (PMN-HIOs). PMN chemoattractants were produced at high levels in infected HIOs (S1 Fig), which could guide PMN transmigration into the lumen of the HIOs. To quantify PMN recruitment into infected HIOs, PMNs were pre-labeled with carboxyfluorescein succinimidyl ester (CFSE) prior to co-culture with HIOs. PMN-HIOs were collected at 8h post-infection (hpi), washed to remove unassociated neutrophils, dissociated into a single cell suspension and the percentage of CFSE-positive cells out of all cells in the HIO was enumerated by flow cytometry (Figs 1A and S2). There was a significant increase in the number of PMNs associated with infected HIOs compared to PBS controls, with approximately 5% of total cells present in PMN-HIOs (including epithelial and mesenchymal cells) staining positive for CFSE (Fig 1A). Immunofluorescent staining for Myeloperoxidase (MPO), as a marker for neutrophils, was performed on paraffin sections to further monitor localization of PMNs within PMN-HIOs. In contrast to PBS-injected controls, MPO-positive cells were observed within the lumen of STM-infected HIOs, confirming that PMNs transmigrate into the HIO lumen during infection (Fig 1B). Since PMNs can act by deploying bactericidal functions, we tested whether PMNs controlled *Salmonella* colonization within the HIO. Although PMNs killed STM in pure PMN cultures, with ~30% of STM killed by 4hpi (S3 Fig), in the HIO model, PMNs did not alter total bacterial burden in the HIOs at 8hpi (Fig 1C). To determine if PMNs were activated, we looked for mobilization of intracellular granules, as indicated by increasing CD63 expression at the cell surface (S4 Fig) and analyzed culture supernatants for production of antimicrobial effectors via ELISA (S5 Fig). Some antimicrobial effectors such as Elafin (PI3), a small cationic peptide secreted at mucosal surfaces [18], Calprotectin (S100A8 and S100A9), Lipocalin-2 (N-GAL), and Beta-Defensin-2 (DEFB4) were produced at higher levels in PMN-HIOs, compared to HIOs alone. However, since *Salmonella* has evolved mechanisms to overcome Calprotectin-mediated immunity and thrive under these conditions; upregulation of these specific antimicrobial effectors is likely insufficient to reduce *Salmonella* colonization in the PMN-HIOs [19,20]. Collectively, these data support that PMNs can migrate into the lumen of the infected PMN-HIO, but do not reduce overall bacterial burden, which largely reflects luminal bacteria.

During intestinal infection, *Salmonella* resides in both the lumen of the intestine and within epithelial cells, and it has been suggested that the intracellular pool of bacteria are important for reseeding the gut lumen to prolong infection and promote fecal shedding [7,21]. To assess what impact PMNs have on intracellular bacterial burden, paraffin sections of *Salmonella-*infected HIOs and PMN-HIOs were stained to detect both epithelial cells and *Salmonella* and the epithelial bacterial burden was quantified by fluorescence microscopy (Fig 1D–1F). This analysis revealed that there were significantly fewer intracellular bacteria in the epithelial lining of PMN-HIOs compared to HIOs alone, indicating that PMNs aid in reducing epithelial cell bacterial burden. Interestingly, we also observed a reduction in epithelial surface-associated bacteria, suggesting that PMNs also reduce STM attachment to further protect the epithelial lining (Fig 1E). Together, these results demonstrate that PMNs contribute to reducing bacterial burden within the epithelial layer.

### PMNs enhance shedding of epithelial cells during *Salmonella* infection

**Fig 2:**
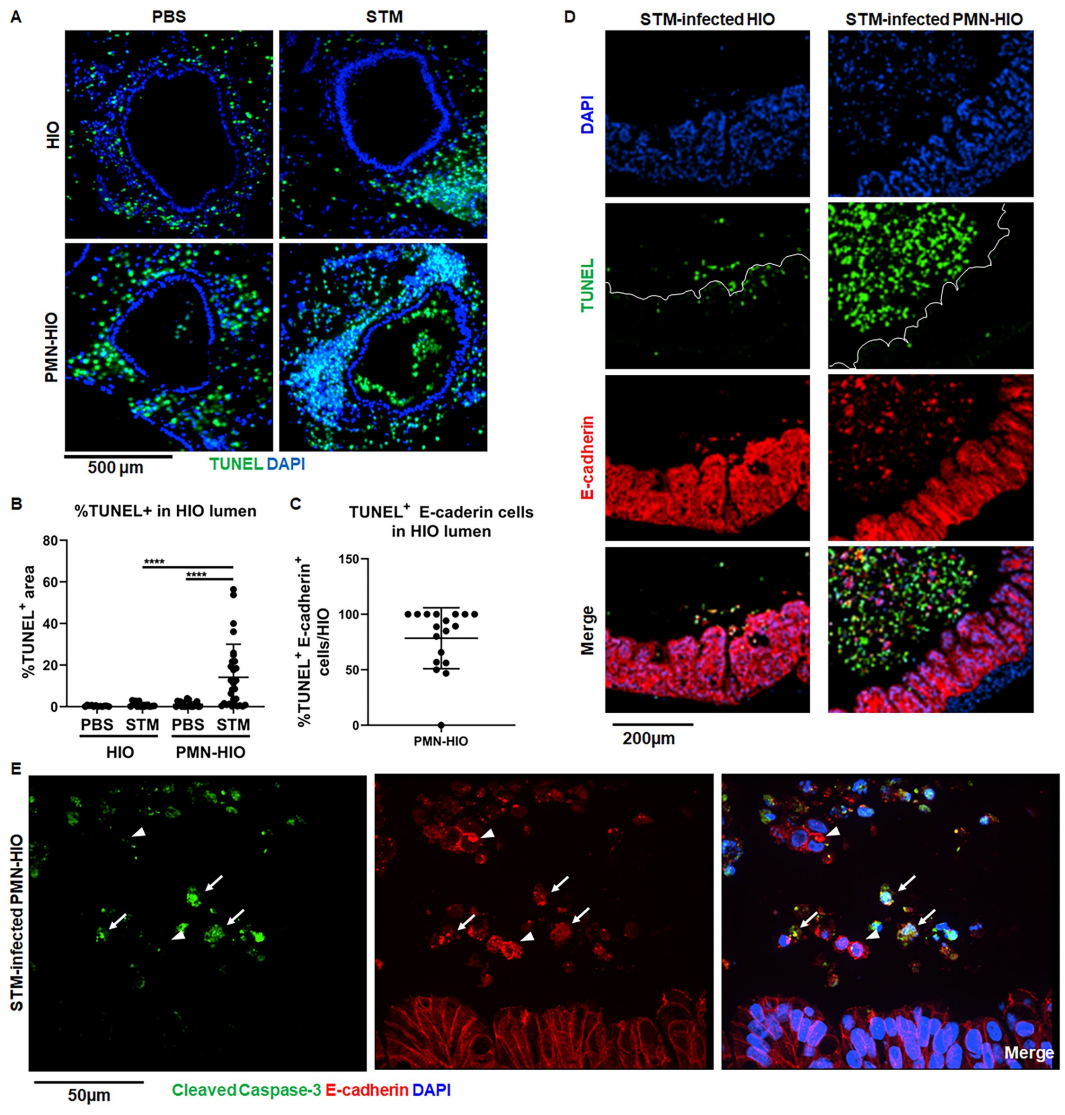
PMNs induce epithelial cell death and shedding during *Salmonella* infection. A. Immunofluorescent images of TUNEL staining of histology sections of HIOs and PMN-HIOs injected with PBS or STM at 8hpi. B. Quantitation of TUNEL positive cells in the lumen of HIOs and PMN-HIOs from (A). Graphs show the mean and SD of HIOs from 2 independent experiments with n>12 HIOs per group. C. Quantitation of percent of TUNEL-positive epithelial cells in HIO lumen. The percentage of TUNEL-positive cells that stained positive for E-cadherin in the HIO lumen were assessed. D. Representative confocal microscopy images of histology sections from STM-injected HIOs or PMN-HIOs at 8h. Sections were co-stained with TUNEL (green), epithelial cell marker E-cadherin (red), and DNA marker DAPI (blue). E. Confocal microscopy images of histology sections of HIOs and PMN-HIOs that were stained for E-cadherin (red), cleaved Caspase-3 (green), and DNA (blue). Arrows point to cleaved Caspase-3 positive epithelial cells whereas arrowheads point to cleaved Caspase-3 negative luminal epithelial cells. Outliers were removed using the ROUT method with Q = 0.1%. Significance was determined via one-way ANOVA with post-Tukey’s test for multiple comparisons where ****p<0.0001.

In addition to measuring a significant reduction in intraepithelial bacterial burden and association of STM with epithelial cells, we observed robust accumulation of DAPI-positive cells in the lumen of STM-infected PMN-HIOs that were negative for the neutrophil-associated marker, MPO (Fig 1B and 1F). Shedding of *Salmonella*-infected cells from the gut via programmed cell death pathways is an important defense mechanism used to protect the host from invasive *Salmonellosis* and helps reduce intestinal bacterial burden to resolve the infection, so we reasoned that PMNs might enhance this process [4–7,22]. To determine whether these luminal cells were dead or dying epithelial cells shed from the epithelial lining, we performed terminal deoxynucleotidyl transferase dUTP nick end labeling (TUNEL) on HIOs and PMN-HIOs microinjected with either STM or PBS. The presence of PMNs induced robust accumulation of TUNEL-positive cells in the lumen of infected HIOs (Fig 2A and 2B). While we also detected a substantial number of TUNEL-positive cells in the mesenchyme this phenotype was present in all conditions, suggesting that this phenotype is likely a result of HIO culture conditions and was not caused by either *Salmonella* or PMNs. Accumulation of luminal TUNEL-positive cells was selectively induced by PMNs during infection, as neither STM-infected HIOs or uninfected PMN-HIOs exhibited this phenotype. Immune stimulation alone was not sufficient to induce this phenotype as there was significantly less accumulation of TUNEL-positive signal in the lumen of LPS-injected PMN-HIOs (S6 Fig). To assess whether these cells were epithelial cells, we stained for the epithelial marker E-cadherin, and found that on average 75% of TUNEL-positive cells in PMN-HIOs were epithelial cells, although this number may be an underestimate since E-cadherin signal appears to decrease following cell extrusion (Fig 2C and 2D). Previously we reported that STM infection in HIOs results in induction of TUNEL-positive cells that are retained in the epithelial layer [23], Here, our results suggest that PMNs enhance shedding of these TUNEL-positive cells from the monolayer as we observed very few TUNEL-positive cells remaining in the epithelial lining of infected PMN-HIOs while many accumulate in the lumen (Figs 2D and S7).

TUNEL staining is classically associated with apoptosis, and since activated PMNs can induce apoptotic processes in lung epithelium [24], we hypothesized that PMNs induce epithelial cell apoptosis to reduce bacteria associated with the epithelial lining. To test our hypothesis, we stained STM-infected PMN-HIO sections for cleaved Caspase-3 as a marker of apoptosis. We found that many, but not all luminal epithelial cells were positive for cleaved Caspase-3 (Fig 2E) suggesting that most shed cells are undergoing Caspase-3 mediated cell death. Noticeably, PMN-induced cell shedding was not restricted to infected cells, as we observed both infected and uninfected cells in the PMN-HIO lumen (S8 Fig). These findings point to a prominent role for PMNs in promoting programmed cell death and epithelial cell shedding during *Salmonella* infection of human intestinal cells.

### Inflammasome activation and IL-1 production is mediated by PMNs during infection

**Fig 3:**
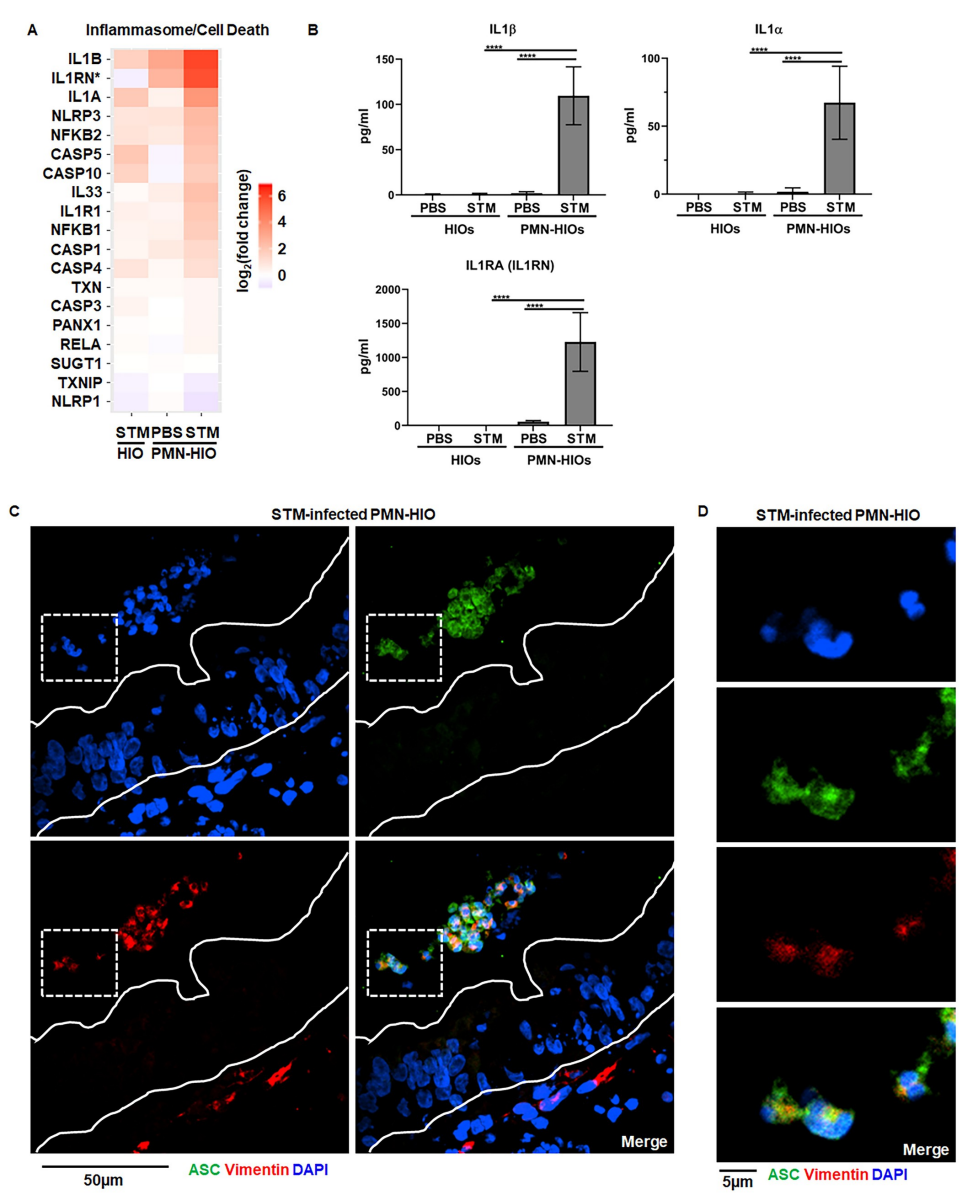
Inflammasome activation and IL-1 production is mediated by PMNs during infection. A. Gene expression data presented as log_2_(fold change) relative to PBS-injected HIOs for genes involved in inflammasome/cell death signaling. All genes are significantly changed from PBS-injected HIOs in at least one condition with p-adjusted value <0.05. B. Cytokine levels in culture media of HIOs and PMN-HIOs were quantified using ELISA. Graphs indicate the mean of n = 4 biological replicates +/- SD from media sampled at 8hpi with 5 HIOs or PMN-HIOs per well. C. Immunofluorescent staining of histology sections of PMN-HIOs. Sections were stained for ASC expression (green), Vimentin (red) to mark PMNs and mesenchymal cells, and DNA (blue) was labeled with DAPI. D. Zoom of (C) showing luminal ASC-positive cells (green) with multilobed PMN nuclei. Statistical significance was determined by 2-way ANOVA where *p<0.05, ***p<0.001, ****p<0.0001.

Inflammasome activation is known to occur in PMNs during bacterial infections [25] and inflammasomes can regulate intestinal inflammation [26]. While activation of noncanonical inflammasomes requiring Caspase-4/5 in human epithelial cells is a hallmark of *Salmonella* infection, prior reports have found in murine intestinal epithelial cells, Caspase-1 together with Caspase-11, protect the epithelial monolayer [6], again highlighting differences in inflammasome engagement between human and murine intestinal cells. However, activation of Caspase-1-dependent inflammasomes in macrophages is critical for effective responses against invading pathogens [27], so we reasoned that neutrophils might also deploy inflammasome machinery in the PMN-HIO model. To assess how PMNs shape inflammasome activation in the PMN-HIO model, we first examined gene level expression data from RNA-seq analysis of HIOs and PMN-HIOs microinjected with PBS or STM (S1 Table). We found that PMNs significantly contributed to upregulation of several genes involved in inflammasome/cell death signaling during STM infection (Fig 3A). While there was weak upregulation of IL-1β and IL-1α in STM-infected HIOs alone, we did not observe significant changes in expression of other mediators or machinery required for NLRP3 inflammasome assembly such as CASP1, NLRP3, or PYCARD (encoding ASC which was not differentially expressed under any condition) (Fig 3A). Consistent with previous reports studying STM-infection in human epithelial models, we did measure a significant increase in Caspase-4 and Caspase-5 transcript levels. In contrast, when PMNs were added to infected HIOs, we observed stronger upregulation of *IL-1* genes and effectors involved in inflammasome activation including the upregulation of *NLRP3* and *Caspase-1* (*CASP1*). To further characterize this phenotype, we collected culture supernatants from HIOs and PMN-HIOs and quantified levels of IL-1 family cytokines during infection (Fig 3B). IL-1β or IL-1α was undetectable in infected HIOs alone; release of these cytokines required the presence of PMNs as IL-1β or IL-1α levels significantly increased in STM-infected PMN-HIOs. These results suggest that PMNs are required for production of IL-1 family cytokines in this infection model, consistent with previous reports that human epithelium is not a major source of IL-1 cytokines [28,29] and that activation of Caspases-3, -4, -5 are not classically associated with IL-1β processing [30]. We observed production of IL-1RA, the IL-1 receptor antagonist which is usually co-expressed with IL-1α/β [31], in infected PMN-HIOs revealing an additional role for PMNs in inducing signaling processes that tune the magnitude of immune activation. To further define which cells within the PMN-HIOs contribute to inflammasome activation and IL-1 processing, paraffin sections of STM-infected PMN-HIOs were stained for ASC, an adaptor protein required for inflammasome assembly (Fig 3C and 3D) [32]. ASC-positive signal was not observed in epithelial cells, consistent with a recent report that *Salmonella*-induced epithelial cell death occurs independently of ASC [9]. Instead, ASC was observed in cells positive for vimentin, a protein expressed by PMNs and mesenchymal cells within the PMN-HIOs. ASC^+^Vimentin^+^ cells were primarily located within the PMN-HIO lumen and closer examination of their nuclear morphology by DAPI staining revealed multi-lobed nuclei. Together, these findings are consistent with a model where PMNs are the primary site of Caspase-1-dependent inflammasome activation and the production of IL-1 family cytokines during STM infection in the PMN-HIO model.

### Caspase-1 and Caspase-3 inhibition reduces epithelial cell shedding and increases association of *Salmonella* with the epithelium

**Fig 4:**
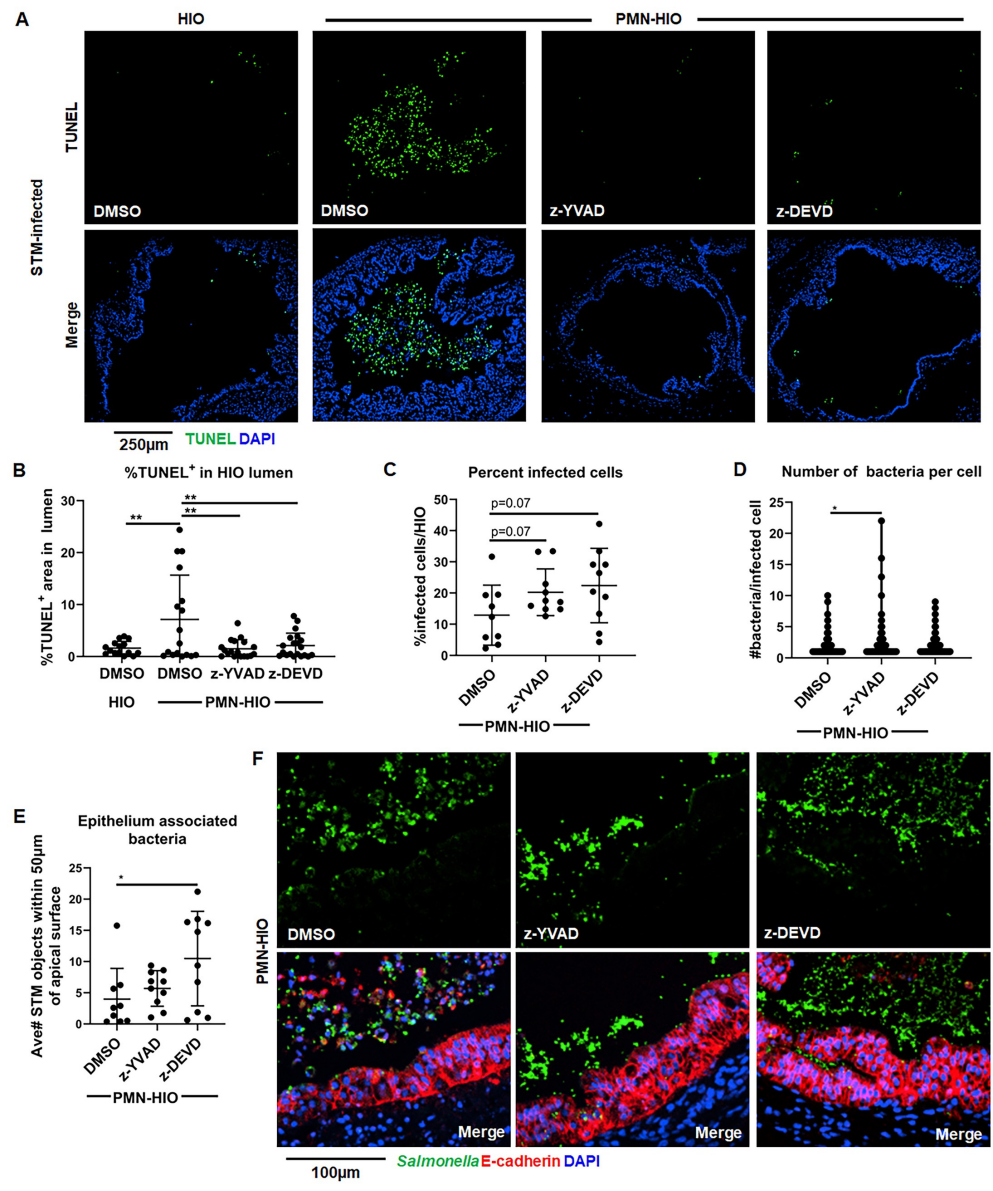
Caspase-1 and Caspase-3 inhibition reduces shedding of infected epithelial cells in the lumen of PMN-HIOs and differentially affect bacterial burden and bacterial association with the epithelium. A. Representative fluorescence microscopy images of TUNEL staining of HIO and PMN-HIO histology sections. HIOs were microinjected with STM and either cultured alone or co-cultured with PMNs in the presence of inhibitors for Caspase-1 (z-YVAD), Caspase-3 (z-DEVD), or DMSO control. B. Quantitation of the percent of lumen filled with TUNEL-positive cells of STM-infected HIOs or PMN-HIOs with indicated treatments. C. Quantitation of the percent of infected cells per HIO based on 3 fields per view per HIO. D. Quantitation of number of bacteria per infected cell in PMN-HIOs based on 3 fields per view per HIO. E. Quantitation of epithelium associated bacteria. Number of bacteria within 50μm of the apical epithelial surface were counted and normalized per 100μm distance. F. Fluorescent microscopy images of STM-infected PMN-HIO histology sections. Samples were stained for *Salmonella* (green), E-cadherin (red), and DAPI (blue). Unless otherwise stated, graphs show the mean +/-SD of n≥ 10 HIOs represented by dots from at least two independent experiments. Outliers were removed using the ROUT method with Q = 0.1%. Significance was determined by one-way ANOVA with post-Tukey’s test for multiple comparisons where *p<0.05, **p<0.01.

Epithelial cell death and shedding serve to reduce bacterial burden in the intestinal epithelium [4–8,22]. To define functional consequences of PMN-induced epithelial cell death on host defense and determine which caspases were involved, we treated PMN-HIOs with selective caspase inhibitors. Accumulation of TUNEL^+^ epithelial cells was monitored in infected PMN-HIOs in the presence or absence of Caspase-1 (z-YVAD-FMK) or Caspase-3 (z-DEVD-FMK) inhibitors. We performed TUNEL staining on paraffin sections from infected PMN-HIOs at 8hpi (Fig 4A and 4B). Both Caspase-1 and Caspase-3 inhibition significantly reduced accumulation of TUNEL^+^ cells in the lumen of infected PMN-HIOs, indicating that PMN-dependent Caspase-1 and -3 activation is required for efficient shedding of epithelial cells. To test how caspase inhibition and reduced shedding affected STM infection, infected PMN-HIOs were stained with an anti-*Salmonella* antibody to quantify the percentage of infected cells, number of bacteria per cell, and epithelium-associated bacteria (Fig 4C–4F). Consistent with our hypothesis that PMNs enhance shedding of epithelial cells through caspase activation, there were greater numbers of bacteria per cell in Caspase-1 inhibitor-treated PMN-HIOs, but surprisingly not in Caspase-3 inhibitor-treated PMN-HIOs (Fig 4D). Caspase-4/5 activation in human epithelial cells contributes to shedding of *Salmonella*-infected cells [22] and our data suggest that PMNs may also enhance epithelial Caspase-4/5 signaling via Caspase-1 activity to increase cell shedding. We also observed a trending increase in the percentage of infected cells upon Caspase-1 inhibition (Fig 4C). In contrast, when infected PMN-HIOs were treated with the Caspase-3 inhibitor, there was a significant increase in epithelium-associated bacteria (Fig 4E). There is some evidence that activated PMNs induce apoptosis of intestinal epithelial cells [10,11,24]; PMN recruitment and activation during *Salmonella* infection in PMN-HIOs may result in enhanced epithelial cell shedding independently of PMN Caspase-1 to increase the rate of cell turnover and thereby reduce the association of bacteria with the apical surface of the epithelium. These data suggest that both Caspase-1 and Caspase-3 inhibition reduce accumulation of dead cells in the PMN-HIO lumen with Caspase-1 activity regulating intraepithelial bacterial burden, while Caspase-3 reduces association of bacteria with the epithelium possibly by stimulating shedding of uninfected epithelial cells. Collectively, our findings implicate PMNs as driver of epithelial cell shedding via two distinct caspase pathways to control *Salmonella* infection in the HIO.

### Caspase-4 regulates accumulation of TUNEL-positive cells in PMN-HIOs

**Fig 5:**
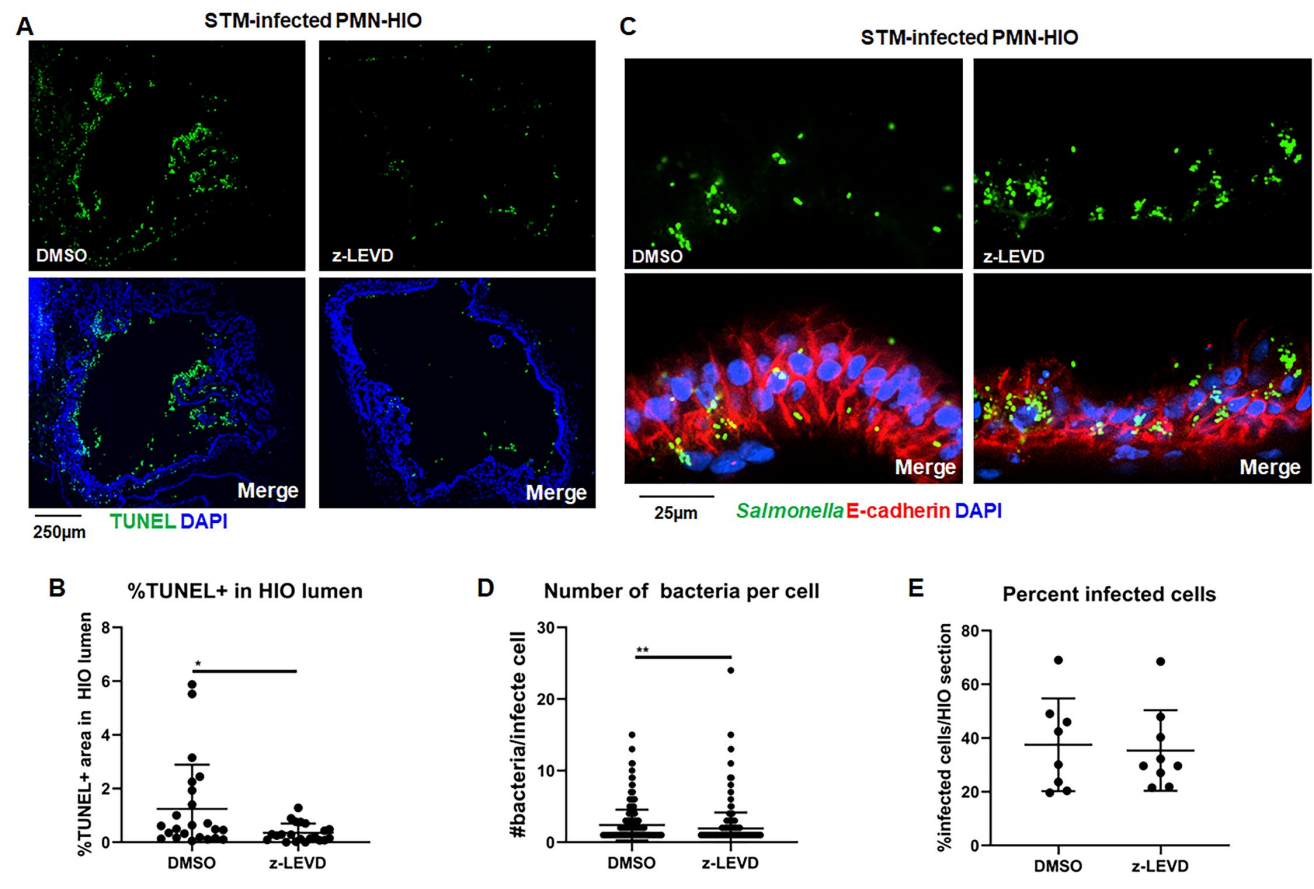
Caspase-4 regulates cell death in STM-infected PMN-HIOs. A. Representative fluorescence microscopy images of TUNEL staining of PMN-HIO frozen histology sections. HIOs were microinjected with STM and co-cultured with PMNs in the presence of inhibitors for Caspase-4 (z-LEVD) (20 μM), or DMSO control. B. Quantitation of the percent of lumen filled with TUNEL-positive cells of STM-infected PMN-HIOs with indicated treatments. C. Fluorescent microscopy images of STM-infected PMN-HIO frozen histology sections. Samples were stained for *Salmonella* (green), E-cadherin (red), and DAPI (blue). D. Quantitation of the number of bacteria per infected cell. E. Quantitation of the percent of infected cells per HIO based on 3 fields per view per HIO. Unless otherwise stated, graphs show the mean +/-SD of n≥ 10 HIOs represented by dots from at least two independent experiments. Outliers were removed using the ROUT method with Q = 0.1%. Significance was determined by unpaired t-test where *p<0.05, **p<0.01.

Caspase-4/5 has an established role for intestinal epithelial cell shedding during *Salmonella* infection [8,22]. We next interrogated the role of these Caspases in the PMN-HIO co-culture model. First, to confirm that Caspase-4/5 were expressed in the HIOs, lysates from PBS or STM-injected HIOs were analyzed by immunoblotting (S9 Fig). Caspase-4 was expressed in both uninfected and infected HIOs, but there was no detectable Caspase-5 (S9A and S9B Fig), even though Caspase-5 could be detected in lysates from a biopsy-derived human intestinal enteroid line [33]. We tested the contribution of Caspase-4 in PMN-mediated epithelial cell shedding during STM infection by treating PMN-HIOs with a Caspase-4 inhibitor (z-LEVD-FMK). Consistent with previous reports [8], Caspase-4 inhibition significantly reduced accumulation of TUNEL-positive cells in the PMN-HIO lumen (Fig 5A and 5B). To test the impact of Caspase-4-mediated cell death on bacterial burden in the epithelial layer, we enumerated bacteria per cell and % infected cells per HIO using immunofluorescence microscopy of infected PMN-HIO histology sections (Fig 5C–5E). STM-infected PMN-HIOs were treated with Caspase-4 inhibitor and stained with anti-*Salmonella* and E-cadherin antibodies. Surprisingly, there was no significant difference in the percent of infected cells with z-LEVD treatment, although there was a modest statistically significant increase in the number of bacteria per cell in inhibitor-treated PMN-HIOs. To confirm that the inhibitor was effective at inhibiting Caspase-4, human monocyte-derived macrophages were stimulated with LPS in the presence or absence of z-LEVD and IL-1β secretion monitored by ELISA, since IL-1β secretion requires Caspase-4 in human monocytes (S9C Fig) [34]. These results suggest that Caspase-4 partially contributes to cell death and control of *Salmonella* burden in the intestinal epithelium of the PMN-HIOs, but clearly implies a reliance on additional mechanisms regulating epithelial cell death.

### IL-1 signaling promotes cell death in STM-infected HIOs

**Fig 6:**
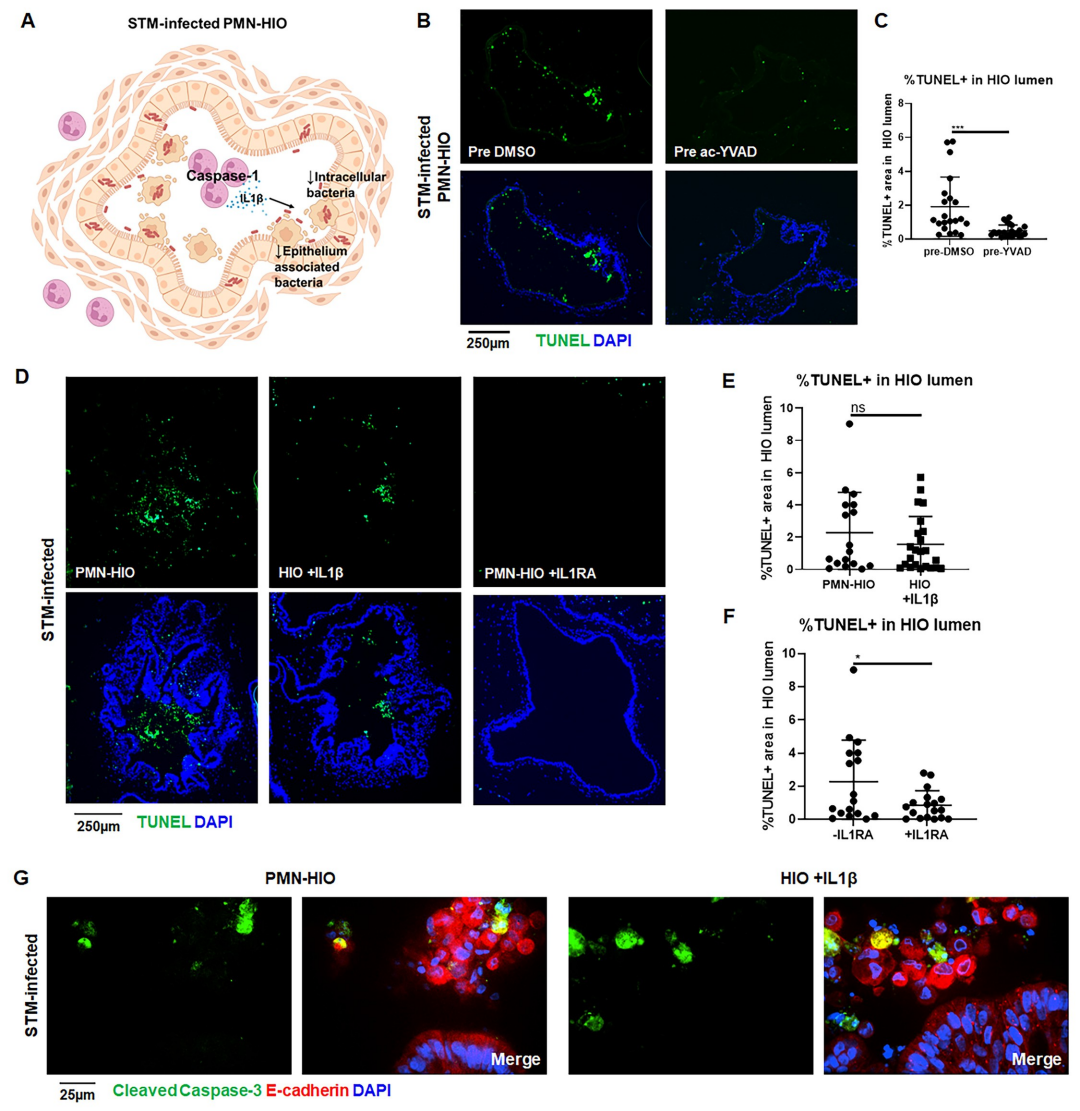
IL-1 signaling mediates shedding of epithelial cells in STM-infected PMN-HIOs. A. Cartoon model showing Caspase-1 activation in PMNs leading to IL-1β production which regulates cell death in STM-infected PMN-HIOs. B. Representative fluorescence microscopy images of TUNEL staining of PMN-HIO frozen histology sections. PMNs were pre-treated with Caspase-1 inhibitor (ac-YVAD) for 1h prior to co-culturing with STM-infected HIOs. C. Quantitation of the percent of lumen filled with TUNEL-positive cells of STM-infected PMN-HIOs with PMNs pretreated with either DMSO or ac-YVAD. D. Representative fluorescence microscopy images of TUNEL staining of HIO or PMN-HIO frozen histology sections. HIOs were microinjected with STM or STM +IL1β for 2h and then where indicated HIOs were co-cultured with PMNs alone or cultured with PMNs and treated with IL1RA (1 μg/ml). E. Quantitation of the percent of lumen filled with TUNEL-positive cells of STM-infected HIOs either co-cultured with PMNs or co-microinjected with recombinant IL1β. F. Quantitation of the percent of lumen filled with TUNEL-positive cells of STM-infected PMN-HIOs treated with or without IL1RA. G. Confocal microscopy images of frozen histology sections of STM-infected HIOs microinjected with recombinant IL1β or STM-infected PMN-HIOs that were stained for E-cadherin (red), cleaved Caspase-3 (green), and DNA (blue). Unless otherwise stated, graphs show the mean +/-SD of n≥ 10 HIOs represented by dots from at least two independent experiments. Outliers were removed using the ROUT method with Q = 0.1%. Significance was determined by unpaired t-test where *p<0.05, ***p<0.001.

Our results led us to hypothesize a role for neutrophil IL1-β in the PMN-HIOs as a crucial signal for effective epithelial cell shedding during *Salmonella* infection (Fig 6A). To further test this model, we pretreated PMNs with a Caspase-1-specific irreversible inhibitor (ac-YVAD-cmk) prior to co-culture with HIOs (Fig 6B and 6C). We observed a significant reduction in the number of TUNEL^+^ cells when PMNs were pretreated with Caspase-1 inhibitor, indicating that Caspase-1 activity in PMNs was necessary for cell shedding in infected PMN-HIOs. Caspase-1 can process pro-IL-1β to generate the active form, but also cleaves other targets. To investigate whether PMN-dependent IL-1β production was necessary and/or sufficient to cause cell death in the HIOs, STM-infected HIOs were stimulated with recombinant IL-1β and TUNEL staining was performed (Fig 6D and 6E). We observed a similar increase in TUNEL^+^ cells in STM-infected HIOs treated with IL-1β without PMNs, compared to the infected PMN-HIOs, demonstrating that IL-1β signaling is sufficient to drive cell death in the absence of PMNs during STM infection. To further test the role of IL-1β signaling, IL-1 receptor was blocked in infected PMN-HIOs using purified recombinant IL-1RA (Fig 6D and 6F). IL1RA treatment reduced accumulation of TUNEL^+^ cells in the STM-infected PMN-HIO lumen, revealing that IL-1 signaling is necessary for cell shedding. Lastly, to determine whether IL-1β treatment in infected HIOs could induce different cell death pathways to recapitulate the phenotype observed in the PMN-HIOs, we stained for cleaved Caspase-3 in STM-infected PMN-HIOs or HIOs treated with recombinant IL-1β (Fig 6G). Immunofluorescence microscopy revealed that IL-1β treatment mirrored the infection in PMN-HIOs with a proportion of shed epithelial cells expressing cleaved Caspase-3. Together these results show that PMN Caspase-1 activity and IL-1 signaling are key drivers of protective cell shedding during *Salmonella* infection of the intestinal epithelium.

## Discussion

Neutrophils (PMNs) dominate the early response to *Salmonella* infection in the gut [35–38], but their functions in regulating human intestinal epithelial cell host defense and infection outcome are not well understood. Here we exploited human intestinal organoids (HIOs) seeded with primary human PMNs, termed PMN-HIOs, to elucidate neutrophil functions in the context of *S*. Typhimurium infection. Human PMNs decreased association of *Salmonella* with the apical surface of the epithelial monolayer and reduced bacterial burden in the epithelium. PMNs caused increased epithelial cell death and promoted extrusion of these cells into the lumen of infected PMN-HIOs. We found that a coordinated program of caspase function controlled PMN-dependent epithelial cell shedding, and that Caspase-1 activation in human PMNs was required. Inhibition of Caspase-1 activity in the PMN-HIOs increased intraepithelial bacterial burden. Additionally, Caspase-3 and Caspase-4 activation in epithelial cells regulated cell death, and Caspase-3 activity reduced association of *Salmonella* with the apical surface. Lastly, we found that IL-1 signaling was necessary and sufficient to drive epithelial cell death and shedding. Thus, we propose a model where activation of Caspase-1 in human PMNs and subsequent IL-1β release enhances protective shedding of epithelial cells from the intestinal barrier.

PMNs are potent mediators of innate immunity, both through their ability to stimulate immune signaling and their microbicidal capacity. PMNs are known to target and kill bacteria that have crossed the intestinal lining to protect the underlying tissue, but it is not well established whether PMNs that transmigrate into the gut lumen also target and kill luminal pathogens [14]. A recent study describing a murine endometrial organoid model found that neutrophils could migrate into the endometrial organoid in response to *Chlamydia muridarum* infection but noted that *Chlamydia* has evolved strategies to restrict neutrophil function [39]. Although we found that primary human PMNs could kill *Salmonella* in vitro, consistent with previous studies, in the more complex environment of the HIO, PMNs did not significantly affect early STM luminal colonization [40]. Instead, our findings clearly indicated that PMNs decreased the population of bacteria within intestinal epithelial cells, pointing to an early role for PMNs in innate immune signaling. Logically, within the environment of the host gut, teeming with microbes, direct bactericidal mechanisms would likely be less effective since neutrophils would be vastly outnumbered [41]. Additionally, while PMNs can be potent killers of invading bacteria, mechanisms used by PMNs to kill pathogens are not selective and therefore PMN activation near the epithelial barrier may be tightly regulated to avoid tissue damage [42]. As one example, we observed robust production of Elafin (S5 Fig), which is annotated as an antimicrobial peptide [43], but can also inhibit neutrophil Elastase to reduce tissue damage caused by neutrophil overactivation [44,45]. Neutrophil Elastase contributes to both intracellular and extracellular antimicrobial functions of PMNs [40,46,47]. Thus, we speculate that expression of host Elafin or other immunoregulatory proteins in the PMN-HIO model may reflect a trade-off in the ability to kill luminal *Salmonella* with protection of epithelial barrier integrity.

Addition of PMNs to the infected HIOs resulted in decreased intracellular bacterial burden, which was unexpected since intracellular bacteria are often considered protected from neutrophil killing. We hypothesized that this result was due to PMN-induced extrusion of infected cells from the epithelial monolayer. The role of the inflammatory Caspases driving cell extrusion differs between human and mouse, with Caspase-4 playing a prominent role in human epithelium in the absence of neutrophils while human epithelial Caspase-1 is largely dispensable [22]. We observed a role for epithelial cell Caspase-4 as well as Caspase-3 in regulating cell death during *Salmonella* infection in the presence of PMNs. Although Caspase-3 and Caspase-4 regulated epithelial cell death during STM infection, inhibition of either Caspase-3 or -4 did not fully reverse the reduction in intracellular bacterial burden that was caused by PMNs. We consider that the enclosed lumen of the HIO may affect infection kinetics or the infection cycle. *In vivo* infections with intestinal flow would make functional effects of cell extrusion on bacterial numbers more apparent since extruded cells would be flushed away. Additionally, the epithelium may have compensatory mechanisms to rid the intestinal lining of bacteria when either Caspase-3 or Caspase-4 are inhibited. Our results highlight that PMNs add a layer of complex regulation controlling cell extrusion and intracellular bacterial burden in a human cell model. Although we could detect low levels of shed epithelial cells in STM-infected HIOs alone consistent with prior studies, this phenotype was markedly enhanced in the presence of PMNs, in a Caspase-1 dependent manner. These data suggest a previously unappreciated role for PMNs in enhancing cell death in *Salmonella*-infected intestinal epithelial cells and implicate Caspase-1 and IL1-β in that process. Moreover, our results reinforce the utility of the HIO model for mechanistic exploration of complex interactions between human epithelial cells and human immune cells.

In STM-infected HIOs, we observed close association of clusters of bacteria with the epithelial surface, which would presumably advantage bacterial pathogens by spatial proximity to the monolayer. Strikingly, the introduction of PMNs into the infected HIO led to dispersal of the bacterial clusters into the HIO lumen, concomitant with a substantial increase in TUNEL+ cells. There are numerous mechanisms by which PMNs can drive epithelial cell death including oxidant production, which can activate apoptotic pathways in the epithelium or NET formation [10,11,24,48]. These possibilities are consistent with our findings that both infected and uninfected epithelial cells are shed from the intestinal monolayer in the infected PMN-HIOs, but not the infected HIOs alone. We found that IL-1β production by PMNs contributes to overall shedding of epithelial cells, which was associated with the dispersal of epithelial-associated bacteria. By increasing epithelial cell shedding, this neutrophil-dependent process contributes to reducing bacterial burden within and associated with the epithelium.

Our findings pointed to PMN Caspase-1 activity as a driver of accumulation of shed epithelial cells in the HIO lumen. Although the importance of Caspase-1, and its target, IL1-β, in PMN activation and antimicrobial functions are not fully appreciated in different physiological contexts, our prior studies demonstrated that inflammasome activation in PMNs occurs during bacterial infection [25]. In vivo, PMN IL-1β was essential for host defense against *Staphylococcus aureus* and was sufficient to clear the bacteria [49]. A recent study also showed that signaling by skin epithelium induced production of IL-1β by PMNs [50]. In the intestine, however, IL-1 family cytokines are associated with other inflammatory pathologies including IBD and colorectal cancer and these studies suggest that uncontrolled IL-1 signaling leads to more severe damage [31]. These reports reveal a pattern where IL-1β production by PMNs may help with immune defense, but must be tightly regulated to prevent too much damage. IL1RA production by intestinal epithelial cells helps tune the inflammation and is consistent with our findings that IL1RA production is increased during infection in the PMN-HIOs, and added IL1RA dampens PMN-mediated cell shedding in the HIO. Our data lead us to propose a model whereby PMNs transmigrate into the inflamed intestine, Caspase-1 is activated and IL-1β released to trigger epithelial cell death and shedding of infected cells to protect the epithelium from ongoing infection. We reason that in the environment of the infected gut, in contrast to infected tissue, the ratio of commensal and pathogenic bacteria to neutrophils may preclude substantive bacterial killing through direct anti-microbial mechanisms. Therefore, signaling mechanisms whereby neutrophils are able to direct protective epithelial responses may more be more advantageous to the host in promoting clearance of intestinal pathogens.

## Materials and methods

### Human Intestinal Organoids (HIOs)

HIOs were generated by the *In Vivo* Animal and Human Studies Core at the University of Michigan Center for Gastrointestinal Research as previously described [51]. Prior to experiments, HIOs were removed from the Matrigel, washed with DMEM:F12 media, and re-plated with 5 HIOs/well in 50μl of Matrigel (Corning) in ENR media ((DMEM:F12, 1X B27 supplement, 2mM L-glutamine, 100ng/ml EGF, 100ng/ml Noggin, 500ng/ml Rspondin1, and 15mM HEPES). Media was exchanged every 2–3 days for 7 days. To ensure that HIO morphology was not impacted by infection, epithelium thickness of HIOs microinjected with either PBS or STM was quantified by measuring epithelial thickness of 3 random region of the HIO on Hematoxylin and Eosin-stained paraffin histology sections (S10 Fig).

### Human Polymorphonuclear Leukocytes (PMNs)

PMNs were isolated from blood of healthy human volunteers as previously described [25]. The purity of PMNs was assessed by flow cytometry using APC anti-CD16 and FITC anti-CD15 antibodies (Miltenyi Biotec); markers characteristic of human neutrophils. PMNs were labeled with cell trace CFSE dye (Thermo Fisher). PMNs were incubated at room temperature for 20 minutes in PBS containing 5 μM CFSE. Cells were washed twice with PBS to remove excess dye and collected by centrifugation. CFSE-labeled PMNs were then co-cultured with STM-infected HIOs or PBS control to monitor the association of PMNs with intestinal epithelial cells. PMN-HIOs were washed twice with PBS to remove unassociated PMNs, mechanically dissociated into single-cell suspension using a 70 μm cell strainer and analyzed on FACSCanto flow cytometer. Percent of CFSE-positive cells were determined using FlowJo software.

### Bacterial growth and HIO microinjection

*Salmonella enterica* serovar Typhimurium SL1344 (STM) was used throughout the manuscript. Bacteria were stored at -80°C in Luria-Bertani (LB, Fisher) medium containing 20% glycerol and cultured on LB agar plates. Individual colonies were grown overnight at 37°C under static conditions in LB liquid broth. Bacteria were pelleted, washed and re-suspended in PBS. Bacterial inoculum was estimated based on OD_600_ and verified by plating serial dilutions on agar plates to determine colony forming units (CFU). The lumen of individual HIOs were microinjected with glass caliber needles with 1μl of PBS or STM (10^5^ CFU/HIO) as previously described [23,52,53] or 1 ng STM LPS (Sigma, Cat#L2262-5MG). Where indicated HIOs were also microinjected with 40 ng/ml recombinant IL-1β (Peprotech, Cat#200-01B). HIOs were then washed with PBS and incubated for 2h at 37°C in ENR media. HIOs were treated with 100 μg/ml gentamicin for 15 min to kill any bacteria outside the HIOs, then incubated in fresh medium +/- PMNs (5 X 10^5^ PMNs/5HIOs/well in a 24-well plate). Where indicated, PMNs-HIOs were treated with the following inhibitors after microinjection: 4 μM of Caspase-1 inhibitor, Z-YVAD-FMK, 4 μM Caspase-3 inhibitor, Z-DEVD-FMK, selective Caspase-1 inhibitor, 20 μM ac-YVAD-cmk, Caspase-4 inhibitor 20 μM z-LEVD-FMK, or 1 μg/ml recombinant IL1RA (Peprotech, Cat#50-399-343).

### Bacterial burden and cytokine analyses

Bacterial burden was assessed per HIO. Individual HIOs were removed from Matrigel, washed with PBS and homogenized in PBS. Total CFU/HIO were enumerated by serial dilution and plating on LB agar. For cytokine analysis, media from each well containing 5 HIOs/well were collected at 8hpi. Cytokines, chemokines, and antimicrobial proteins were quantified by ELISA at the University of Michigan Cancer Center Immunology Core.

### Immunofluorescence staining and microscopy

HIOs were fixed with 10% neutral formalin for 2 days and embedded in paraffin. Histology sections (5μm) were collected by the University of Michigan Cancer Center Histology Core. Sections were deparaffinized and antigen retrieval was performed in sodium citrate buffer (10mM sodium citrate, 0.05% Tween 20, pH 6.0). For frozen sections, after fixation in 10% neutral formalin, HIOs were incubated with 20% sucrose for 3 days prior to embedding in OCT. Samples were stored at -80°C until sectioning. Histology sections (10μm) were collected using a cryostat. Sections were brought to room temperature and OCT was removed with a PBS wash prior to staining. For both paraffin and frozen sections, samples were permeabilized with PBS+ 0.2% Triton X-100 for 30 min, then incubated in blocking buffer (PBS, 5% BSA, and 10% normal goat serum) for 1h. Primary antibodies; anti-E-Cadherin (BD Biosciences, clone 36), anti-MPO (Agilent, clone A0398), anti-Vimentin (DSHB, Cat# AMF-17b), anti-ASC (Cell Signaling, Cat#13833), anti-CD63 (Invitrogen, clone MEM-259), and anti-cleaved Caspase-3 (Cell Signaling, Cat# 9661) were added to the histology sections in blocking buffer overnight at 4°C. Goat anti-mouse and anti-rabbit secondary antibodies conjugated to Alexa-488, Alexa-594 or Alexa-647 were used according to manufacturer’s instructions (Thermo Fisher) for 1h RT in blocking buffer. DAPI (Thermo Fisher) was used to stain DNA. Bacteria were stained using anti-*Salmonella* Typhimurium FITC-conjugated antibody (Santa Cruz, Cat# sc-52223). Sections were mounted using coverslips (#1.5) and Prolong Diamond or Prolong Glass Antifade Mountant (Thermo Fisher). Images were taken on Olympus BX60 upright compound microscope, Nikon A1 confocal microscope or Nikon X1 Yokogawa spinning disc confocal microscope and processed using ImageJ and quantitation was performed in ImageJ or CellProfiler.

### TUNEL assay

Apoptosis was analyzed by fluorescence microscopy using *In Situ Cell Death Detection Kit* (Roche) or CF594 TUNEL Assay Apoptosis Detection Kit (Biotium) according to the manufacturers’ protocols. Histology sections were permeabilized using Proteinase K (20μg/ml) or 0.2% Triton X-100 in PBS and blocked using PBS+ 5% BSA. Sections were stained with primary antibodies overnight at 4°C in blocking buffer and then were incubated in the terminal deoxynucleotidyl transferase end labeling (TUNEL) buffer for 1h at 37°C. Slides were washed with PBS and incubated with fluorescent conjugated secondary antibodies. Sections were then counterstained with DAPI to label DNA. To quantify the TUNEL signal in the HIOs, the percent of the HIO lumen filled with TUNEL+ cells was quantified using ImageJ software.

### Immunoblotting

HIOs were lysed with 0.1% NP40 lysis buffer containing protease inhibitor cocktail (Roche). HIO lysates were centrifuged, Laemmli sample buffer was added, and samples were heated at 95°C for 15 min. Proteins were separated by SDS-PAGE on gradient gels (4–20%, Bio-Rad), transferred onto nitrocellulose membranes and blocked with blocking buffer: PBS containing 0.05% Tween-20 and 5.0% dry milk. Membranes were incubated with primary antibodies in blocking buffer overnight at 4°C. After washing with PBS, membranes were incubated with secondary LI-COR antibodies for 1h at room temperature, washed 3 times with PBS and visualized using the Odyssey Infrared Imaging System (Li-Cor Biosciences). The following antibodies were used: anti-Caspase-4 antibody (4B9) (Santa Cruz, Cat# sc-56056), anti-Caspase-5 (D3G4W) antibody (Cell Signaling, Cat#46680), IRDye 800CW Goat anti-Mouse IgG antibody (LI-COR, Cat# P/N 925–32210), and IRDye 680RD Goat anti-Rabbit IgG antibody (LI-COR, Cat# 925–68071).

### RNA sequencing and analysis

Total RNA was isolated from 5 HIOs per group with a total of 4 replicates per condition using the mirVana miRNA Isolation Kit (Thermo Fisher). The quality of RNA was confirmed, ensuring the RNA integrity number (RIN)> 8.5, using the Agilent TapeStation system. cDNA libraries were prepared by the University of Michigan DNA Sequencing Core using the TruSeq Stranded mRNA Kit (Illumina) according to the manufacturer’s protocol. Libraries were sequenced on Illumina HiSeq 2500 platforms (single-end, 50 bp read length). All samples were sequenced at a depth of 10.5 million reads per sample or greater. Sequencing generated FASTQ files of transcript reads that were pseudoaligned to the human genome (GRCh38.p12) using kallisto software [54]. Transcripts were converted to estimated gene counts using the tximport package [55] with gene annotation from Ensembl [56].

### Gene expression and pathway enrichment analysis

Differential expression analysis was performed using the DESeq2 package [57] with *P* values calculated by the Wald test and adjusted *P* values calculated using the Benjamani & Hochberg method [58].

### Quantification and statistical methods

RNA-seq data analysis was done using RStudio version 1.1.453. Plots were generated using ggplot2 [59] with data manipulation done using dplyr [60]. Other data were analyzed using Graphpad Prism 9. Statistical differences were determined using statistical tests indicated in the figure legends. The mean of at least 2 independent experiments were presented with error bars showing standard deviation (SD). *P* values of less than 0.05 were considered significant and designated by: *P < 0.05, **P < 0.01, ***P < 0.001 and **** *P* < 0.0001.

## Supporting information

S1 Table RNA-seq data at 8hpi of STM-infected HIOs and PMN-HIOs. Significant genes with log_2_(fold change) and adjusted p-value relative to PBS-injected HIOs.
(XLSX)

S1 Fig HIOs produce PMN chemoattractants. Quantitation of protein levels in culture media of HIOs and PMN-HIOs microinjected with PBS or STM for 8h measured by ELISA. Graphs indicate the mean of n = 4 replicates +/-standard deviation. Significance was determined by 2-way ANOVA where *p<0.05, ***p<0.001, ****p<0.0001.
(TIF)

S2 Fig PMN transmigrate into HIOs during STM infection. Representative flow plots of PMN transmigration during infection. PMNs were prelabeled with CFSE prior to co-culture with HIOs. PMN-HIOs were then dissociated into single cells and run through the flow cytometer to quantify %CFSE+ cells.
(TIF)

S3 Fig PMNs kill STM. PMN bactericidal activity against *Salmonella* was quantified by enumerating CFU at 4h in the presence of PMNs relative to bacteria cultured alone. Results are from n = 4 independent experiments with PMNs isolated from blood of different donors.
(TIF)

S4 Fig CD63 relocalizes to PMN cell surface during STM infection in PMN-HIOs. Immunofluorescent image of PMN-HIO lumen during STM infection showing CD63 localized to cell periphery as a marker of PMN activation. Arrow points to an example of CD63 localized to the cell surface of an MPO-positive cell.
(TIF)

S5 Fig The antimicrobial response is intact in PMN-HIOs. Quantitation of antimicrobial protein levels in culture media of HIOs and PMN-HIOs microinjected with PBS or STM for 8h measured by ELISA. Graphs indicate the mean of n = 4 replicates +/-standard deviation. Significance was determined by 2-way ANOVA where *p<0.05, ***p<0.001, ****p<0.0001.
(TIF)

S6 Fig LPS injection does not induce robust TUNEL accumulation in PMN-HIOs. A. Immunofluorescent images of TUNEL staining of frozen histology sections of HIOs and PMN-HIOs injected with PBS or 1ng LPS at 8h. B. Quantitation of TUNEL positive cells in the lumen of PMN-HIOs from (A). Graph shows the mean and SD of HIOs from 2 independent experiments with n>10 HIOs per group. Outliers were removed using the ROUT method with Q = 0.1%. Significance was determined via unpaired t-test where **p<0.01.
(TIF)

S7 Fig TUNEL signal in epithelial cells retained in monolayer. A. Immunofluorescent images of TUNEL staining of paraffin histology sections of HIOs and PMN-HIOs injected with STM at 8hpi. B. Quantitation of nuclear TUNEL intensity of cells retained in the epithelial lining from (A). Graphs show the mean and SD of HIOs from 2 different batches of HIOs with n>4 HIOs per group. Outliers were removed using the ROUT method with Q = 0.1%. Significance was determined via unpaired t-test where ****p<0.0001.
(TIF)

S8 Fig Some but not all extruded cells are infected with *Salmonella*. Immunofluorescent staining of STM-infected PMN-HIOs stained for *Salmonella* (green), E-cadherin (red), and DAPI (blue). Arrowheads point to uninfected extruded cells.
(TIF)

S9 Fig Caspase-4 and -5 expression in HIOs and inhibition. A. Western blot measuring Caspase-4 levels in HIO lysates microinjected with PBS or STM. Blot was probed for Actin as a loading control. B. Western blot measuring Caspase-5 levels in HIO lysates that were microinjected with PBS or STM. Lysates from human intestinal enteroids (HIEs) were included as a positive control for the antibody. The blot was also probed for Actin as a loading control. C. IL-1β ELISA from supernatants of human monocyte derived macrophages stimulated with LPS for 6h. Cells were treated +/-z-LEVD.
(TIF)

S10 Fig HIO epithelium thickness does not change with infection. Epithelium thickness was quantified using hematoxylin and eosin (H&E) stained histology sections. 3 regions at random per image were measured from ≥7 HIOs. Significance was determined by unpaired t-test.
(TIF)
