# Supplementary figures and images for: Human neutrophil IL1β directs intestinal epithelial cell extrusion during Salmonella infection
Source: PLoS Pathog. 2022 Oct 3;18(10):e1010855. doi: 10.1371/journal.ppat.1010855 (PMC9578578; doi:10.1371/journal.ppat.1010855)

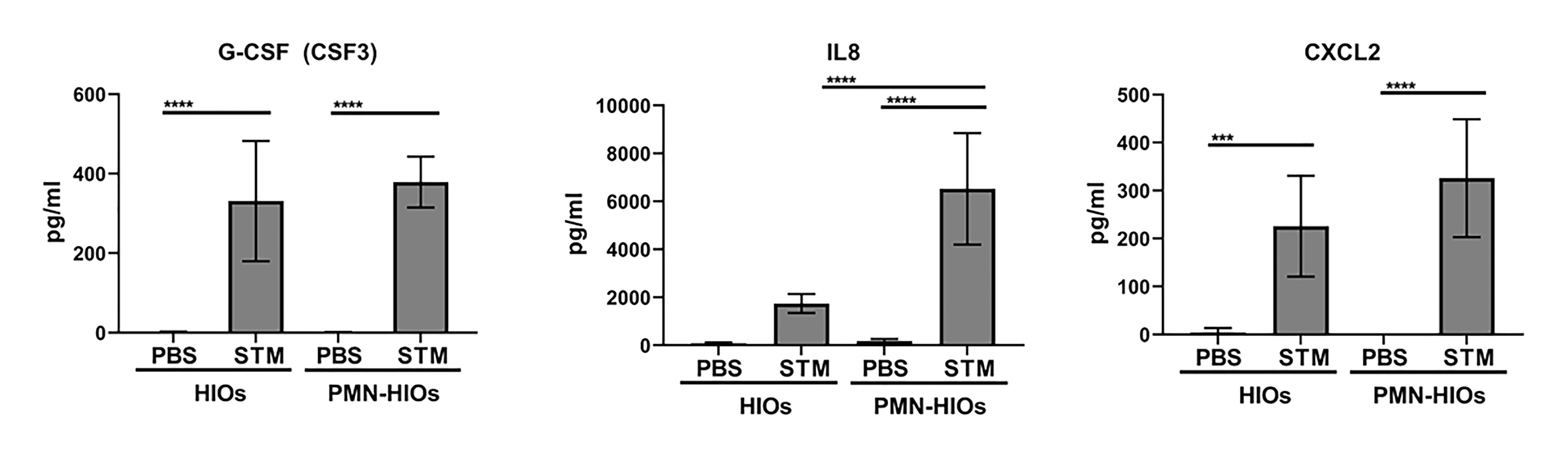

Supplement: S1 Fig — Quantitation of protein levels in culture media of HIOs and PMN-HIOs microinjected with PBS or STM for 8h measured by ELISA. Graphs indicate the mean of n = 4 replicates +/-standard deviation. Significance was determined by 2-way ANOVA where *p<0.05, ***p<0.001, ****p<0.0001. (TIF) [file ppat.1010855.s002.tif]

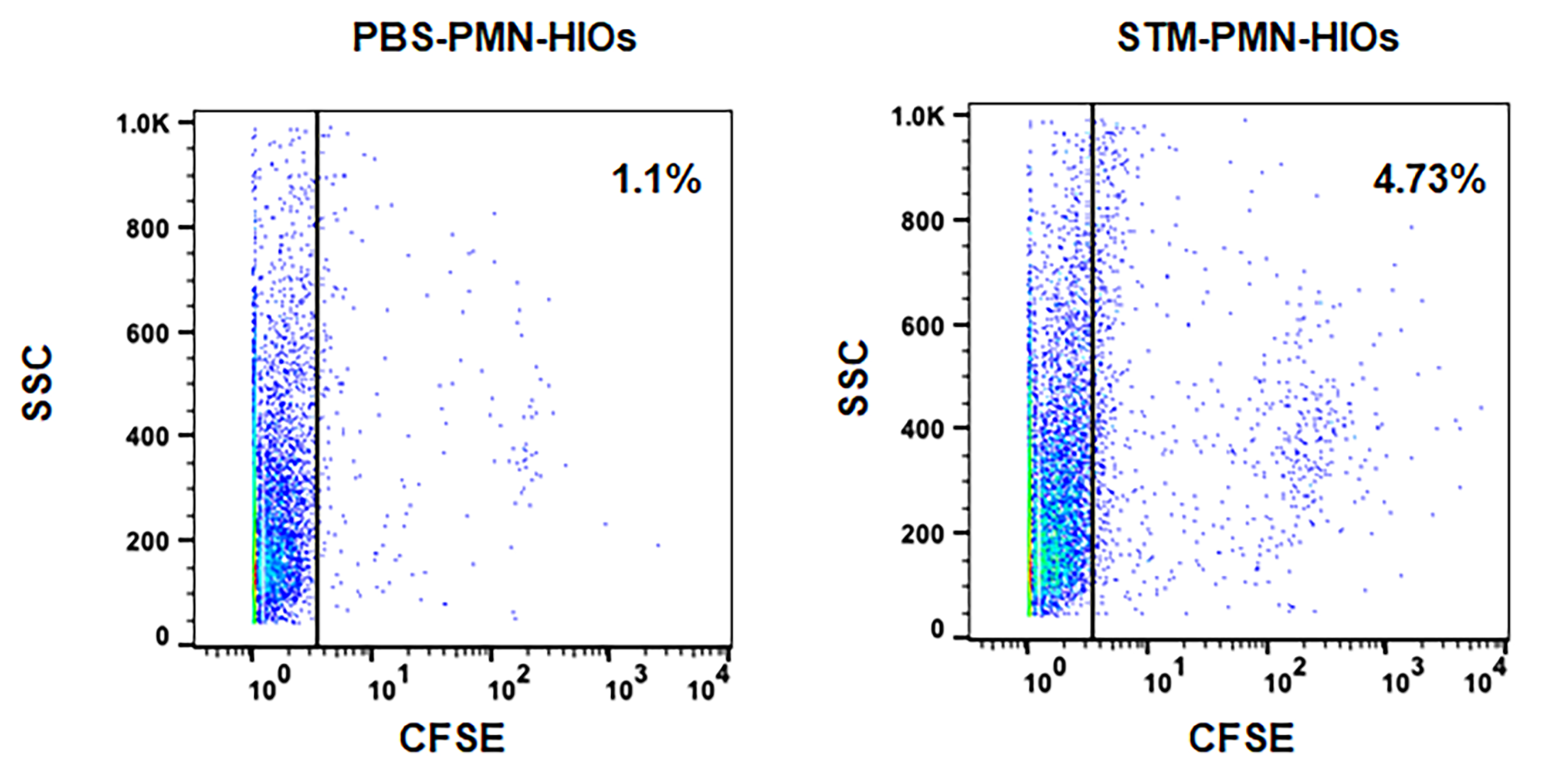

Supplement: S2 Fig — Representative flow plots of PMN transmigration during infection. PMNs were prelabeled with CFSE prior to co-culture with HIOs. PMN-HIOs were then dissociated into single cells and run through the flow cytometer to quantify %CFSE+ cells. (TIF) [file ppat.1010855.s003.tif]

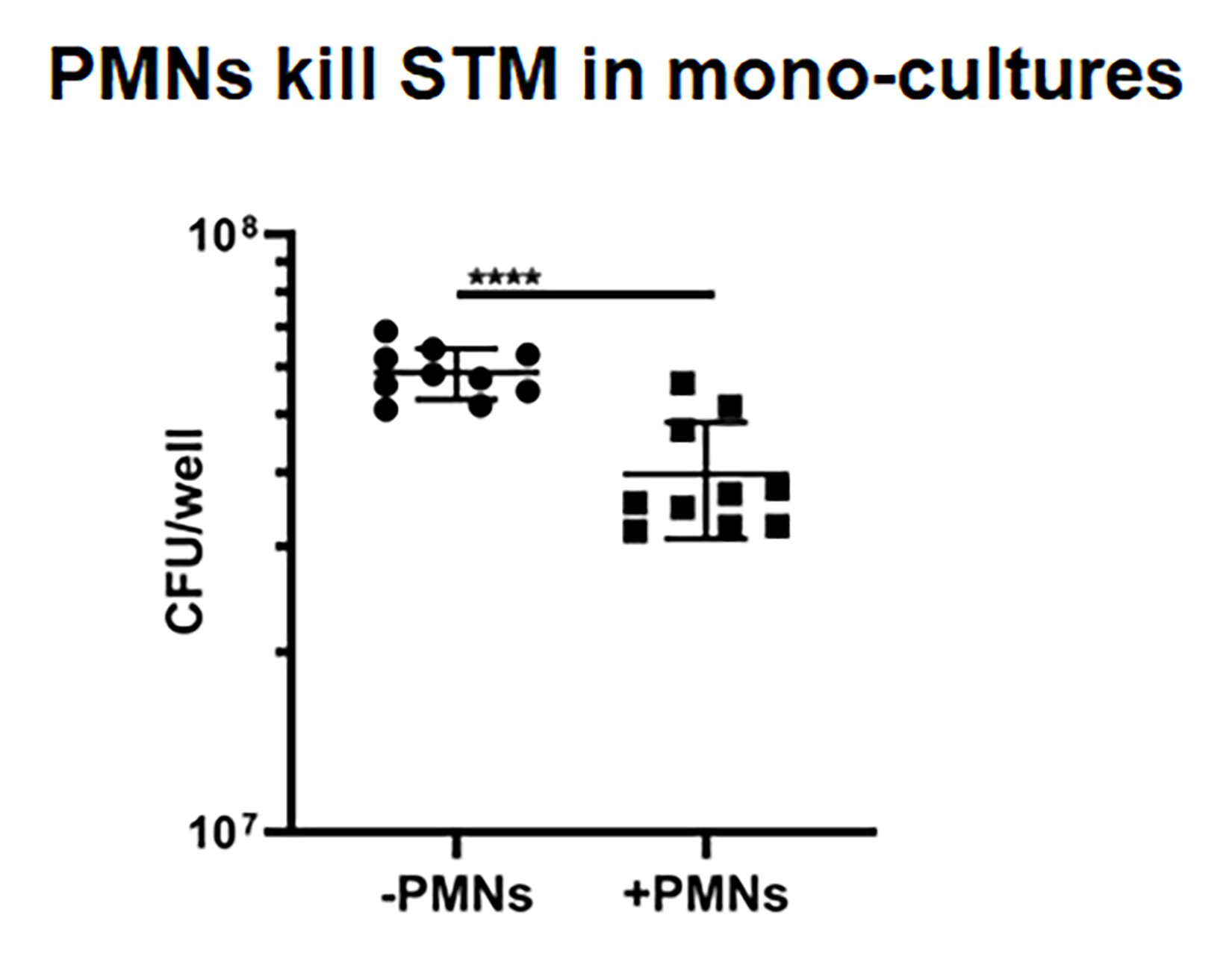

Supplement: S3 Fig — PMN bactericidal activity against Salmonella was quantified by enumerating CFU at 4h in the presence of PMNs relative to bacteria cultured alone. Results are from n = 4 independent experiments with PMNs isolated from blood of different donors. (TIF) [file ppat.1010855.s004.tif]

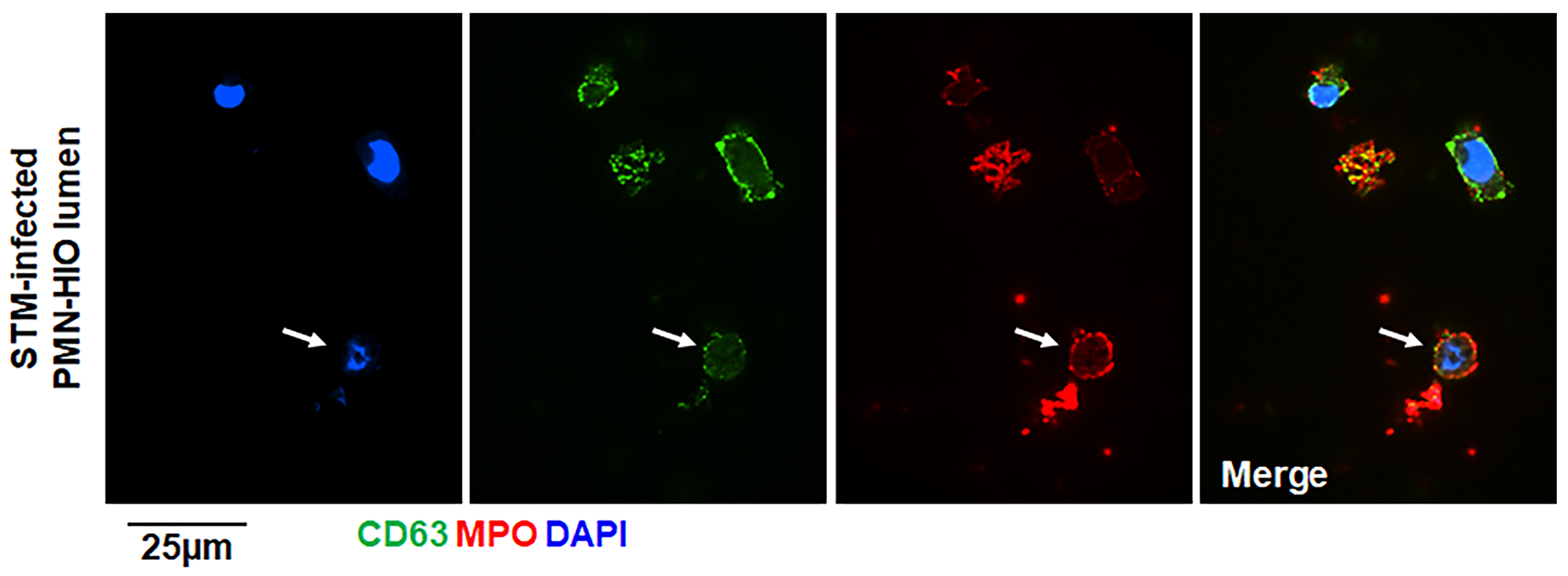

Supplement: S4 Fig — Immunofluorescent image of PMN-HIO lumen during STM infection showing CD63 localized to cell periphery as a marker of PMN activation. Arrow points to an example of CD63 localized to the cell surface of an MPO-positive cell. (TIF) [file ppat.1010855.s005.tif]

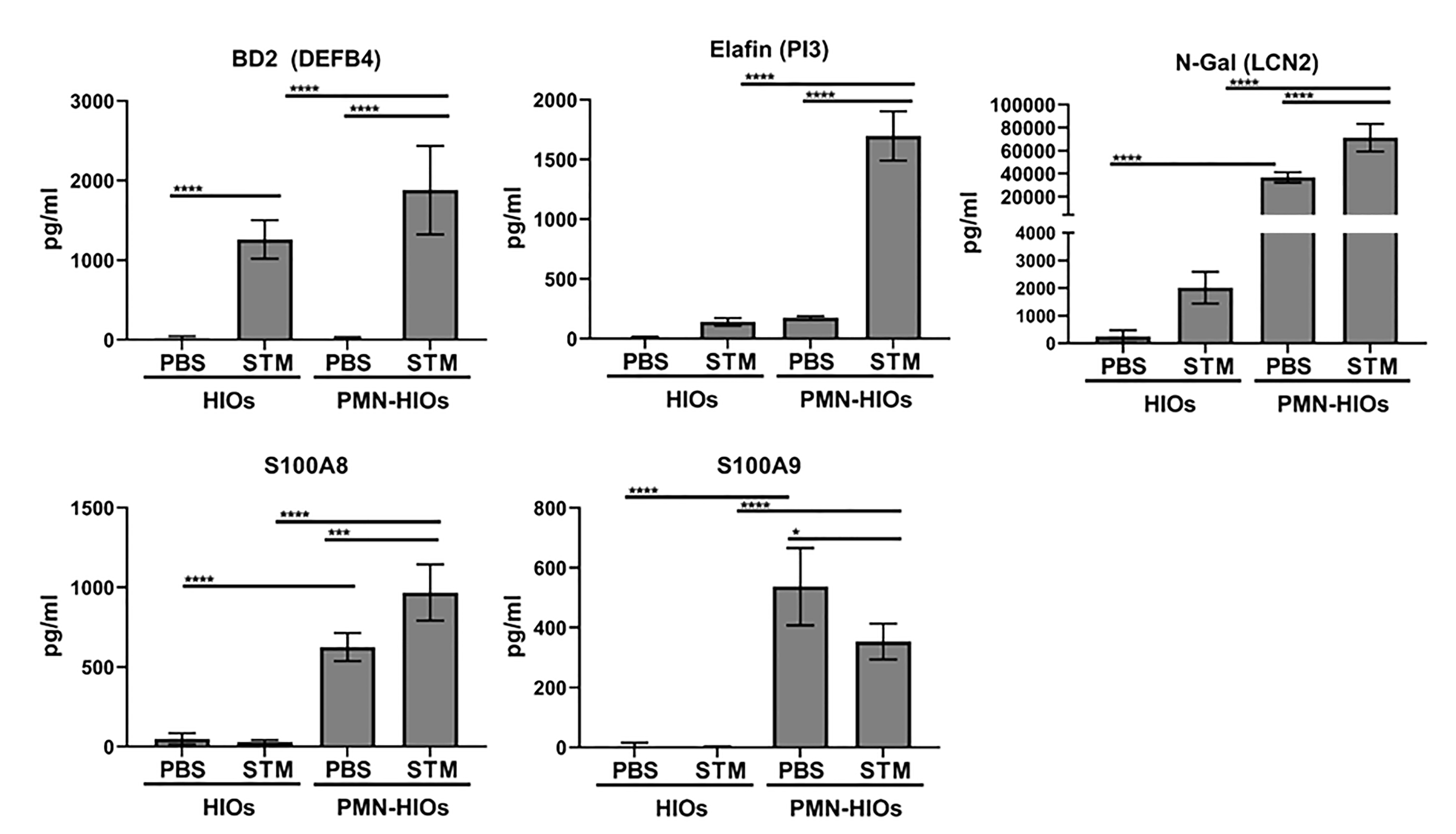

Supplement: S5 Fig — Quantitation of antimicrobial protein levels in culture media of HIOs and PMN-HIOs microinjected with PBS or STM for 8h measured by ELISA. Graphs indicate the mean of n = 4 replicates +/-standard deviation. Significance was determined by 2-way ANOVA where *p<0.05, ***p<0.001, ****p<0.0001. (TIF) [file ppat.1010855.s006.tif]

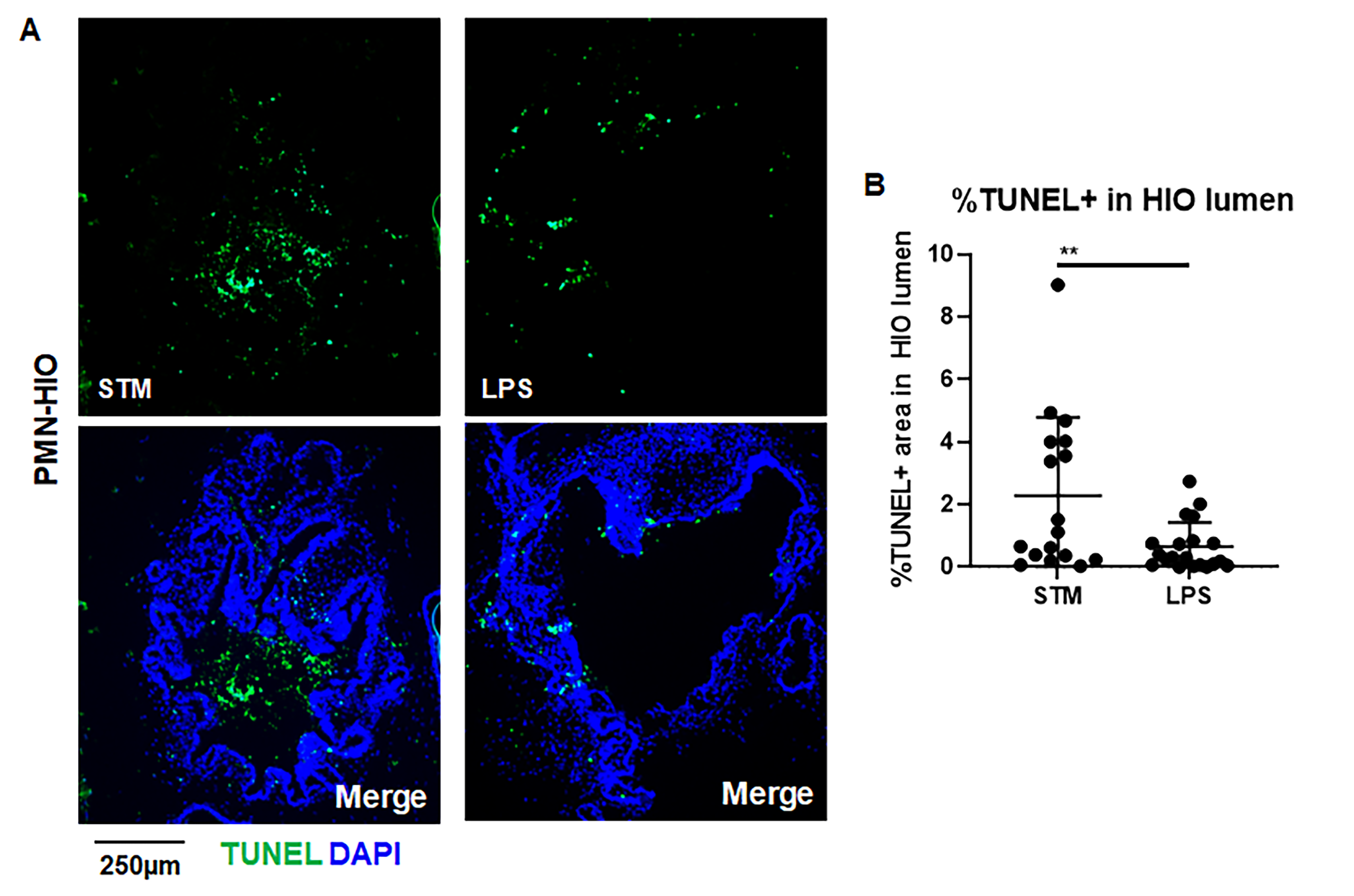

Supplement: S6 Fig — A. Immunofluorescent images of TUNEL staining of frozen histology sections of HIOs and PMN-HIOs injected with PBS or 1ng LPS at 8h. B. Quantitation of TUNEL positive cells in the lumen of PMN-HIOs from (A). Graph shows the mean and SD of HIOs from 2 independent experiments with n>10 HIOs per group. Outliers were removed using the ROUT method with Q = 0.1%. Significance was determined via unpaired t-test where **p<0.01. (TIF) [file ppat.1010855.s007.tif]

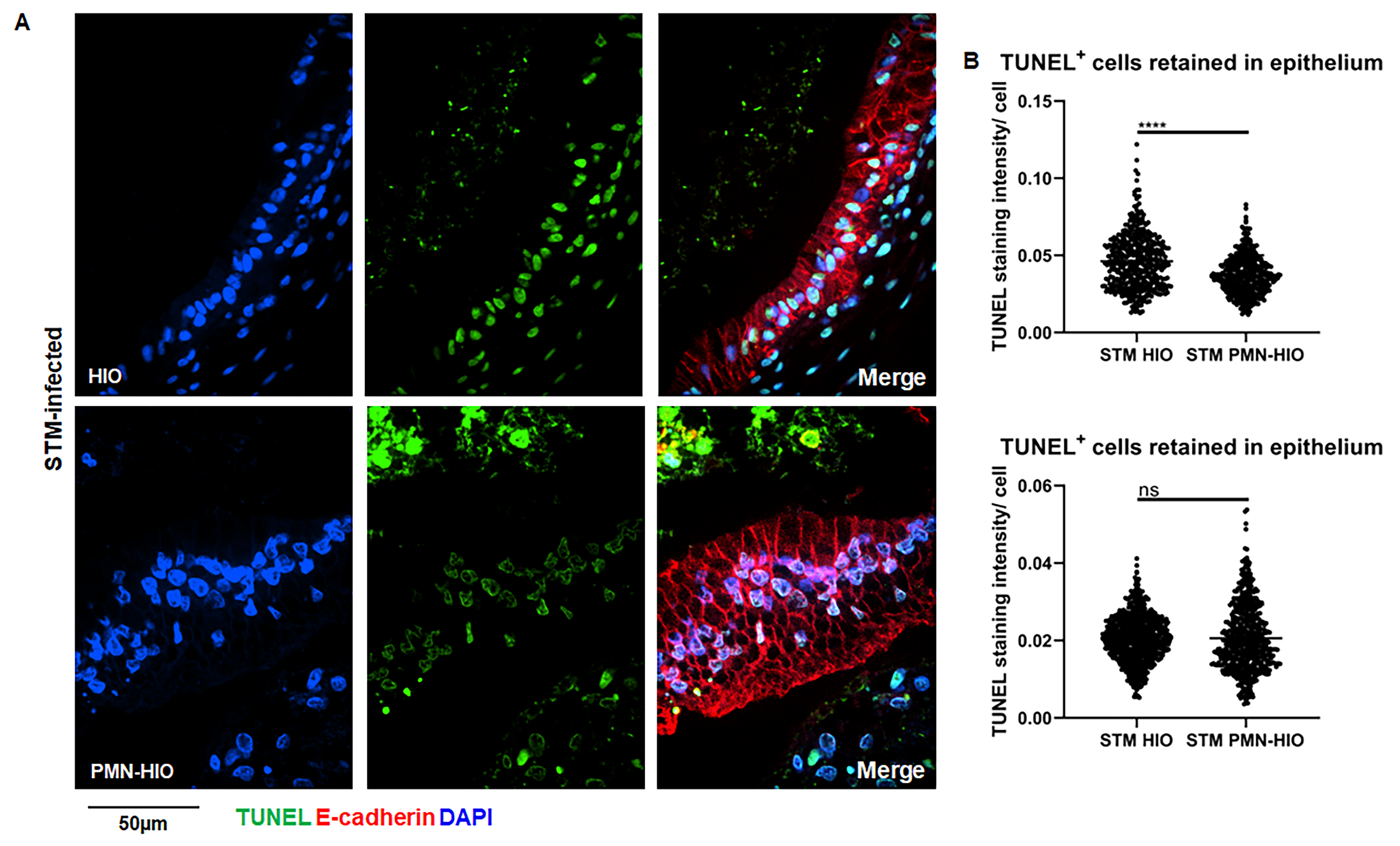

Supplement: S7 Fig — A. Immunofluorescent images of TUNEL staining of paraffin histology sections of HIOs and PMN-HIOs injected with STM at 8hpi. B. Quantitation of nuclear TUNEL intensity of cells retained in the epithelial lining from (A). Graphs show the mean and SD of HIOs from 2 different batches of HIOs with n>4 HIOs per group. Outliers were removed using the ROUT method with Q = 0.1%. Significance was determined via unpaired t-test where ****p<0.0001. (TIF) [file ppat.1010855.s008.tif]

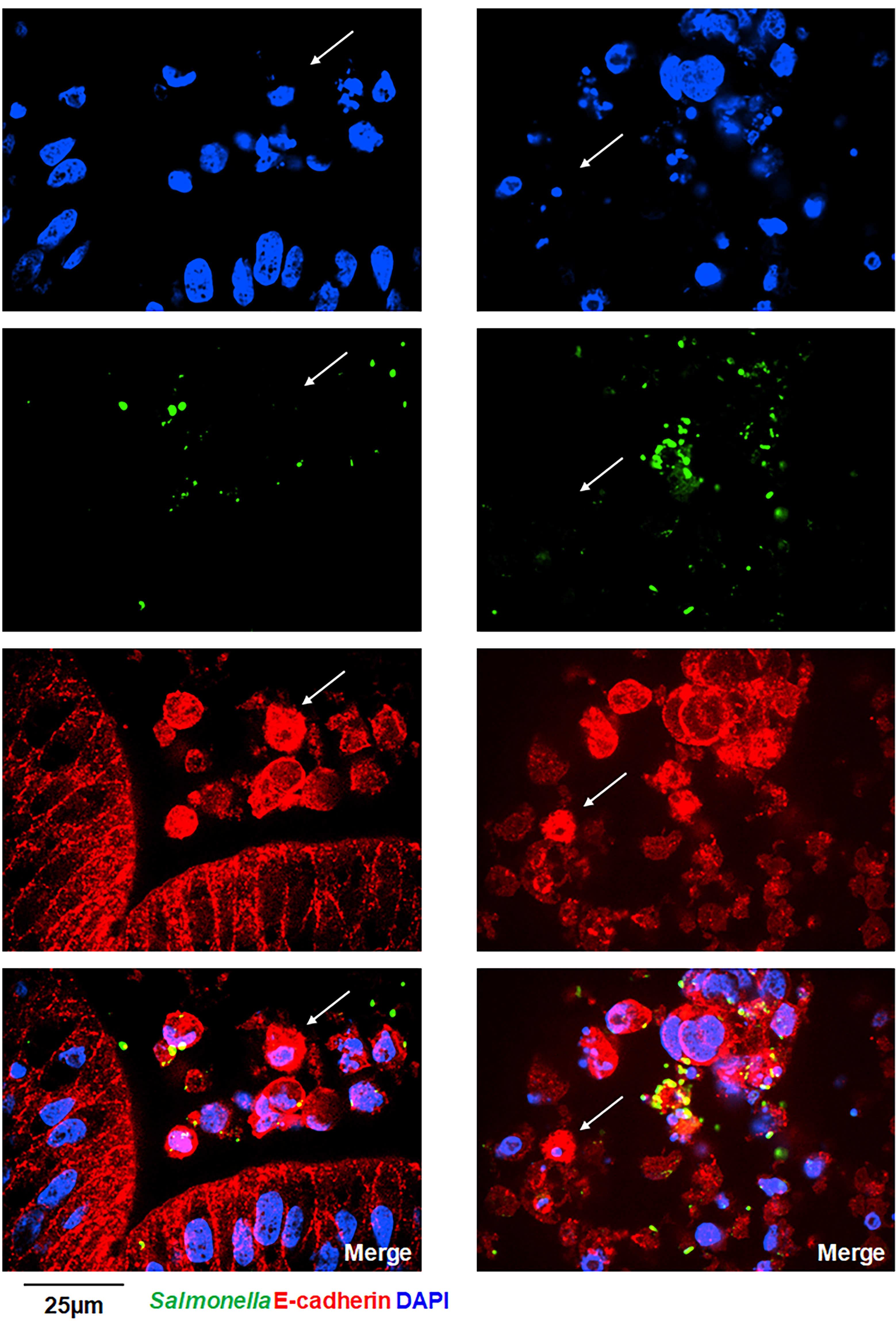

Supplement: S8 Fig — Immunofluorescent staining of STM-infected PMN-HIOs stained for Salmonella (green), E-cadherin (red), and DAPI (blue). Arrowheads point to uninfected extruded cells. (TIF) [file ppat.1010855.s009.tif]

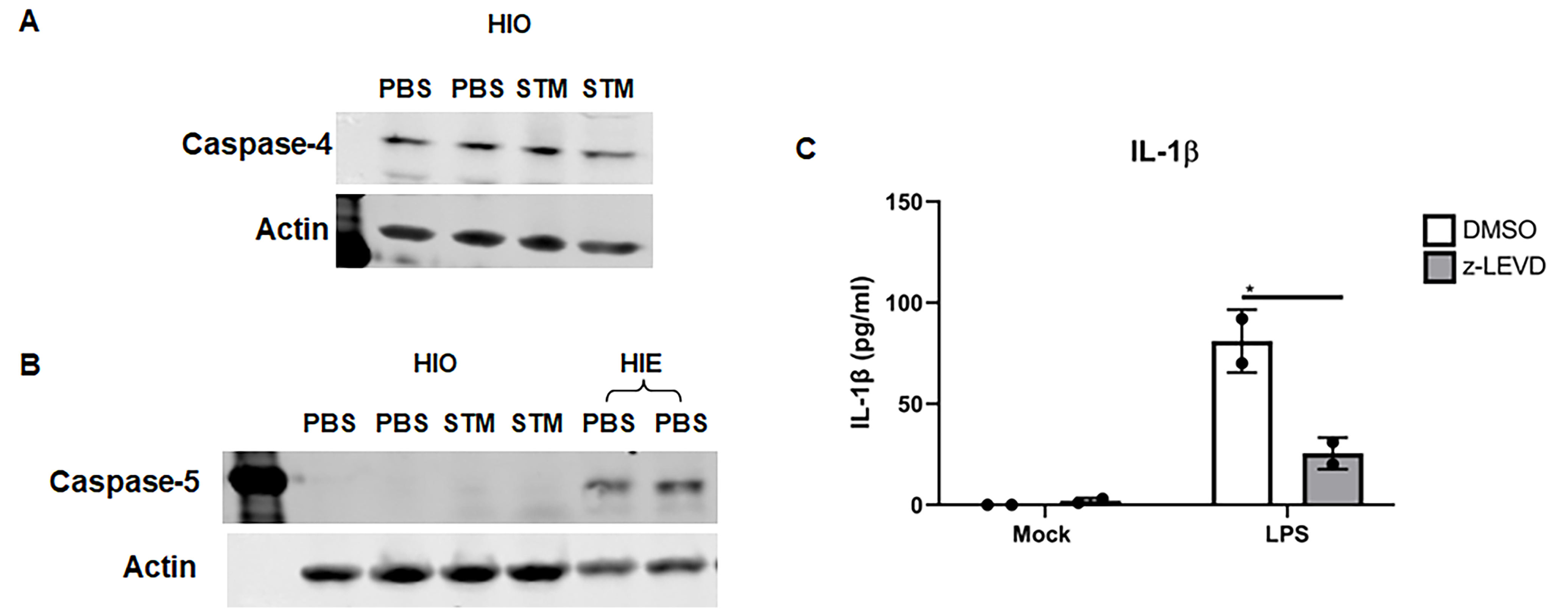

Supplement: S9 Fig — A. Western blot measuring Caspase-4 levels in HIO lysates microinjected with PBS or STM. Blot was probed for Actin as a loading control. B. Western blot measuring Caspase-5 levels in HIO lysates that were microinjected with PBS or STM. Lysates from human intestinal enteroids (HIEs) were included as a positive control for the antibody. The blot was also probed for Actin as a loading control. C. IL-1β ELISA from supernatants of human monocyte derived macrophages stimulated with LPS for 6h. Cells were treated +/-z-LEVD. (TIF) [file ppat.1010855.s010.tif]

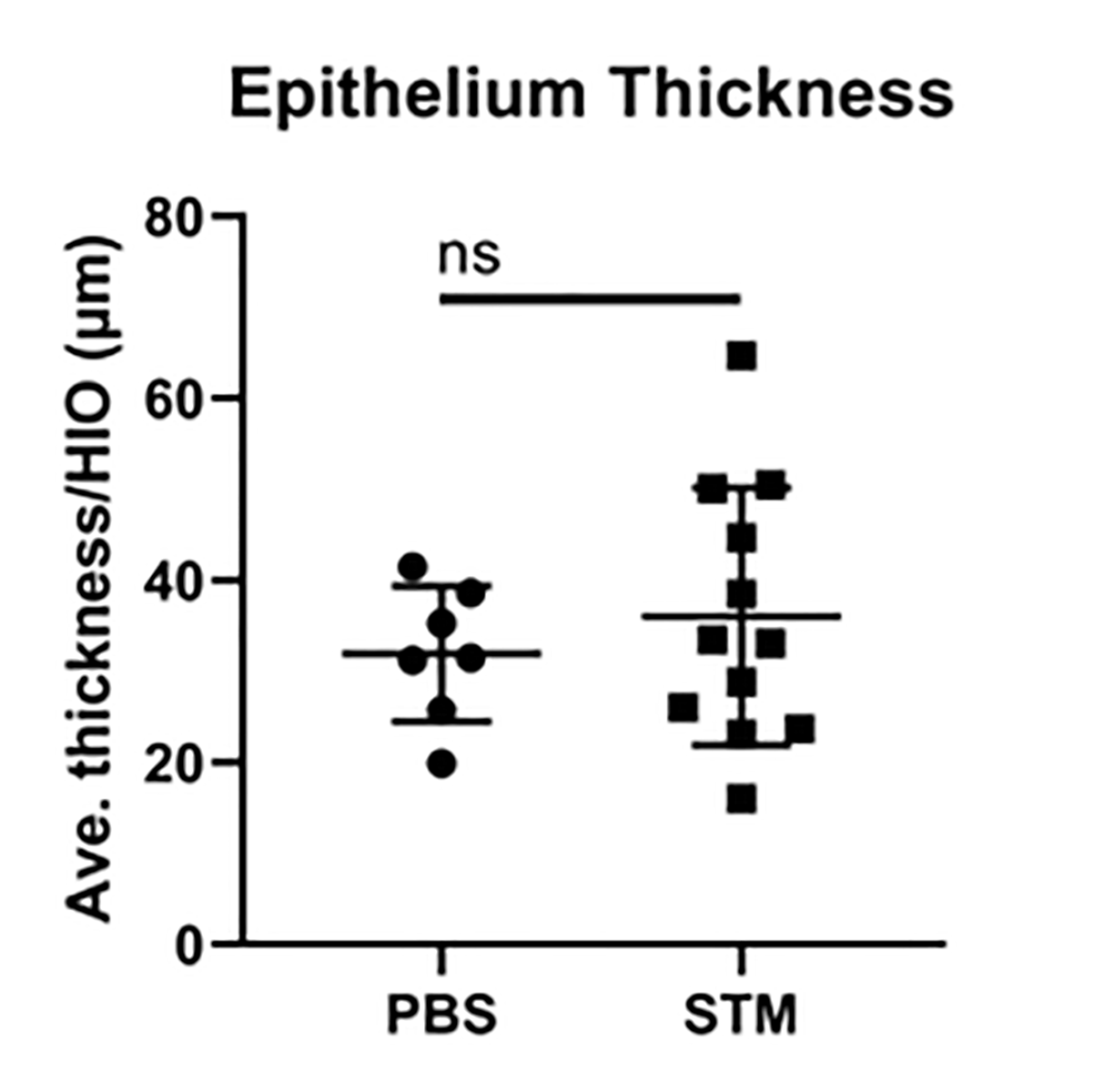

Supplement: S10 Fig — Epithelium thickness was quantified using hematoxylin and eosin (H&E) stained histology sections. 3 regions at random per image were measured from ≥7 HIOs. Significance was determined by unpaired t-test. (TIF) [file ppat.1010855.s011.tif]
