# Supplementary material for: Human neutrophil IL1β directs intestinal epithelial cell extrusion during Salmonella infection
Source: PLoS Pathog. 2022 Oct 3;18(10):e1010855. doi: 10.1371/journal.ppat.1010855 (PMC9578578; doi:10.1371/journal.ppat.1010855)
Supplement: Attachment — Submitted filename: POINT_BY_POINT_REPLY_125751_1_rebuttal_2425419_rfsdp9.docx [file ppat.1010855.s012.docx]

**Manuscript number:** RC-2022-01384

**Corresponding author(s):** Mary O’Riordan and Basel Abuaita

## 1. General Statements [optional]

We appreciated the positive feedback and helpful suggestions from the reviewers that pointed to a need for more clarity regarding the central focus of the study. Our goal was to take an unbiased approach to evaluating the role of neutrophils during *S.* Typhimurium (STM) infection of human intestinal epithelial cells (IEC), using human intestinal organoids as a model. An abundance of data point to important inflammatory roles for neutrophils during STM infection of human intestine but the critical mechanisms involved have not been fully elucidated. New data now included in the revised manuscript provide strong support for human PMN-derived IL1-beta as a driver of epithelial cell shedding in STM-infected HIOs, consistent with known differences in local inflammation between human and mouse infection, and this is the focus of the current study. Our data did not support a significant role for human neutrophils in controlling luminal bacterial numbers, but instead the primary human PMNs robustly stimulated epithelial cell responses that led to decreased intraepithelial bacteria. Several recent studies have suggested that caspase-1 is not a critical inflammasome component during STM infection of IEC, which instead use non-canonical inflammasomes, including caspases-4 and -11. Our data point to a human neutrophil-intrinsic function for caspase-1 and IL1-beta that contributes to the inflammatory tone of the intestinal milieu early in STM infection.

## 2. Point-by-point description of the revisions

Reviewer #1

*Major comments:*

*Some important links are missing to fully support the mechanistic model proposed:*

*1- PMN activity*

*The authors may strengthen their evidence of PMN activities presented in lines 135 to 143 and in Fig.S2 and S3. In particular, the authors claim that PMNs form NETs in PMN-HIOs but the evidence displayed are limited. In fact, Fig S2 shows the same condition and same staining as Fig 1B but the MPO-positive structures are different. Clarification in the text or the figure would be welcome. Besides, as the authors insist on the relevance of NETs in the discussion, it seems that a clear demonstration and characterization of these structures in the PMN-HIO model would highly benefit the manuscript.*

While we commented on NETs in our original manuscript, our conclusions do not rely on the presence or absence of NETs. We have therefore removed the NET data and the reference to NETs. While NETs are potentially interesting in the context of intestinal infection, we understand the reviewer's concern about NETs and anticipate that a more quantitative characterization of NETs may be challenging given the structure and variability of the PMN-HIOs.

*Regarding the analyses of the culture supernatants (Fig.S3), only 3 out of the 5 displayed datasets are commented on in the text. The data obtained for BD2 and N-Gal should be either commented or removed from the figure. The author further suggests that Elafin expression in presence of PMN may restrict PMNs' ability to kill Salmonella. Repeating the experiment displayed in Fig S1 in the presence of Elafin as well as in the presence of the supernatant extracted from HIOs and PMN-HIOs would clarify the potential inhibition of PMN killing capacity in the PMN-HIO model*.

We now include a sentence on the antimicrobials BD2 and N-GAL to the text (line 135-136). Elafin is one of many molecules that could potentially affect the ability of PMNs to kill *Salmonella.* We repeated the experiments in S3 Fig with recombinant Elafin. There was a very weak effect on killing in the presence of Elafin, however Elafin can also kill *Salmonella* directly, complicating interpretation of these experiments. We have now added a sentence in the Discussion to speculate that Elafin is one example of how the epithelium may inhibit the ability of PMNs to kill (line 366-372). These data are not central to our main conclusions and are only intended to provide context to the reader about possible explanations for why PMNs can kill *Salmonella* directly, but do not significantly alter total bacterial numbers in the HIO model.

*The author proposed that infected and uninfected cells are extracted from the epithelium due to PMN activation, suggesting that Salmonella infection of epithelial cells is only indirectly involved in cell shedding. This is an interesting hypothesis that could be tested by measuring cell shedding in a non-infected but PMN-activated (for instance with PMA) PMN-HIO model. This would clarify further the role of PMN in controlling epithelial response to the infection*.

We tested this possibility by microinjecting LPS into the lumen of PMN-HIOs (S6 Fig). There was significantly less TUNEL+ signal in LPS-injected PMN-HIOs compared to STM-infected PMN-HIOs, suggesting that active *Salmonella* infection is required for shedding of both infected and uninfected cells in the presence of PMNs**.**

*2- Specificity of RNA-seq profiling:*

*The authors analyzed the transcriptomic profiling of PMN-HIOs and HIOs infected or not. While these experiments bring to light an interesting difference in inflammasome/cell death transcriptomic programs at the scale of the co-culture model, it is not possible to conclude from which cell type these transcriptomic shifts emerge. To clarify this, the authors stain the co-culture for ASC and observe that ASC-positive cells are PMNs. They conclude that PMNs are most likely the primary site of caspase-1 dependent production of IL1. While their model is theoretically consistent, more direct proofs are necessary to conclude on the cell-type specific transcriptomic program during infection of PMN-HIO and could be obtained by FACS sorting of the cells prior to RNA-seq, for instance using MPO to detect PMNs and E-cadherin to detect epithelial cells.*

We now provide evidence that pretreating PMNs with an irreversible Caspase-1 inhibitor before co-culturing with STM-infected HIOs prevented accumulation of luminal TUNEL+ cells (Fig 6B,C). Additionally, IL-1β treatment in the absence of PMNs recapitulated the cell death phenotype of the infected PMN-HIOs (Fig 6D,E) suggesting Caspase-1 activity in PMNs and IL-1β production are necessary for epithelial cell death in the PMN-HIOs.

*3- Roles of cytokine*

*After showing an increased expression/release of IL1 and IL1RA in infected PMN-HIOs, the authors move on to testing the role of caspases on cell shedding. Yet, they do not test the impact of IL1 and IL1RA on cell shedding. As, according to their proposed model, IL1 is acting upstream of caspase-1 to promote cell shedding, testing cell shedding in infected PMN-HIOs in the presence of an IL1 inhibitor would clarify that link. The author also proposed that the decrease of IL33 in PMN-HIOs compared to HIOs could be due to PMN processing, which would give an additional role to PMNs in controlling the epithelial response to infection. In the context of this manuscript, it would be highly relevant to test this hypothesis by measuring the rate of cleaved IL-33*.

We now provide data to address these questions about IL-1 signaling. HIOs were microinjected with recombinant IL-1β during STM infection and PMN-HIOs were also treated with IL1RA during STM infection. Cell shedding was measured under these conditions in Fig. 6D-F. Cell shedding was dependent on IL-1 signaling and the model has been updated to reflect this.

We also concentrated supernatants from STM-infected HIOs and PMN-HIOs, probed for cleaved IL33 via western blot and did see some cleavage. However, without being able to block this process it is not possible to conclude what role cleaved IL33 has during infection in the PMN-HIO and IL-1β seems to be sufficient to drive the cell shedding phenotype. Since the status of IL33 is not central to our conclusions, we have removed these data from the manuscript.

*4- Roles of caspase*

*The interpretations of the role of Caspases to restrict bacteria burden are unclear and should be revised (see also minor comment). It appears that both Caspase-1 and Caspase-3 are necessary for efficient cell shedding (Fig4B), Caspase-1 (but not Caspase-3) decreases intraluminal bacteria burden (Fig4C) and Caspase-3 (but not Caspase-1) decreases epithelium-associated bacteria (Fig4D). To reconcile these observations with the hypothesis that cell shedding is responsible for the decrease of intraluminal and epithelium-associated bacterial burden, one may propose that caspase-3 (but not caspase-1) induces cell shedding of mainly non-infected cells (possibly bacteria-associated) and caspase-1 (but not caspase-3) induced cell shedding of infected cells. This could be tested by measuring the % of infected extruded cells upon caspase inhibitor treatments. In addition, these data don't allow to propose that Caspase-3 activation happens downstream of Caspase-1 as suggested by the authors in their abstract figure*.

It is difficult to accurately quantify the percent infected cells that are extruded since both infected and uninfected cells are extruded into a luminal space full of bacteria, which may associate with uninfected cells post-extrusion. However, we did observe cells positive for cleaved Caspase-3 when HIOs were treated with IL-1β leading us to infer that Caspase-1 mediated cytokine signaling through IL1R can trigger downstream Caspase-3 activation (Fig. 6G). We have expanded the Discussion to talk about differing roles of Caspases on bacterial burden and association with the epithelium (lines 374-397).

*Minor comments:*

*The majority of the points listed below can be addressed with further analyses of pre-acquired data sets:*

*Fig1E/1F/4D: each green dot is not likely to be individual bacteria but rather a cluster of bacterium (based on their size). So the y-axis in Fig 1E and Fig4D should not be #STM.*

Y-axis labels have been changed to #STM objects

*Fig2A: Variations in organoid size and epithelial thickness can be observed between figures. In particular, in Fig 2A, the HIO seems much younger than the other ones displayed in the manuscript.*

There is considerable natural variability between HIOs and between batches, a phenomenon observed by many HIO researchers (Hofer et al. Nature Reviews Materials 2021). HIOs were all treated the same way prior to infection, and based on our extensive observations, epithelial thickness does not correlate significantly with a particular experimental condition, as we now show in S10 Fig.

*Line 176 to 178, the authors mentioned the TUNEL+ cells in the mesenchyme but rule out the possibility that this phenotype could be infection or PMN-dependent because it is observed in the different conditions. As the picture displayed in Fig2A suggests high differences in the number of TUNEL+ cells in the mesenchyme under the 4 tested conditions, the authors should still quantify this phenomenon (possibly in the supplementary).*

This is likely an artifact of culturing and not due to the infection or PMNs. There is variability between HIO batches in the amount of TUNEL signal in mesenchymal cells (for example HIOs in Fig 4A and 5A have very low or no TUNEL positivity in the mesenchyme).

*"DAPI" should be written in blue.*

This has been corrected.

*Fig2C: Could the authors comment on the % of E-cadherin cells that are also TUNEL+? Is it 100%?*

On average about 75% of TUNEL+ cells are E-cadherin+. We think that this may be an underestimate because E-cadherin staining intensity decreases in many cells after shedding. This is commented on in the text (lines 178-179).

*Fig 2D: The point made on lines 182 to 186 that HIOs contain TUNEL + cells retained in the epithelial lining in the absence of PMNs is not very strongly supported by Fig 2D. Quantification of the number of intraepithelial TUNEL+ cells in the 4 compared conditions would make a more solid case.*

We quantified TUNEL intensity in epithelial cells retained in the monolayer (S7 Fig). We do note that there is some variability in this phenotype that correlates with different batches of HIOs**.**

*Fig2E: This experiment should be completed with a quantification of the percentage of TUNEL+ cells that are also cleaved caspase3-positive. The data, as currently displayed, do not prove that the cells negative for cleaved caspase 3 are apoptotic cells and thus do not support the sentence "suggesting that multiple forms of cell death were occurring in the PMN-HIO" (line 194).*

Cells negative for cleaved Caspase-3 that are TUNEL+ may be undergoing some other form of cell death that is not Caspase-3 dependent, such as necrosis. This possibility is consistent with the decreased TUNEL signal observed upon inhibition of Caspase-4 (Fig 5A,B)**.** However, we have reworded our conclusion to identify more clearly what the data indicate, and where we are drawing inferences.

*Fig3A: "IL1RN" should be changed for "IL1RA (IL1RN)" for consistency with Fig 3B.*

The heatmap shows gene expression data so IL1RN is more consistent with the gene nomenclature. However, we have added an asterisk to the label on the heatmap, along with a sentence in the figure legend to elucidate.

*Fig 4C: The authors should provide the percentage of infected cells rather than the number of bacteria per cell (this information can be included in supplementary).*

Percent infected cells has been moved to Fig 4C and the number of bacteria per cell has been moved to Fig 4D**.**

*FigS2: The different thicknesses of the epithelial layer observed between PBS and STM panels suggest a difference in scale. This may be double-checked by the authors.*

The images are scaled similarly – as noted earlier (S10 Fig), there is considerable natural variability between HIOs that is not correlated with any experimental condition in this study.

*Line 197-199, the authors claimed that uninfected cells may be observed in the cell lumen. This seems hard to observe/conclude at this resolution. The authors may show a non-infected cell at higher magnification*.

We have added higher magnification images, uninfected cells are indicated with white arrows in S8 Fig.

*Discussion: Some important points should be added to the discussion. In particular, what is the fate of intracellular salmonellae after cell shedding? Can the bacterium survive cell apoptosis and burst out of the cell to re-infect the epithelium or be transferred to phagocytic cells during the clearance of intraluminal apoptotic cells?*

*Previous studies showed that cytosolic hyper-replication could fuel cell shedding. The importance of bacterial load in PMN-induced cell shedding could be discussed*.

We have expanded the discussion to elaborate on what may happen to shed cells. One useful feature of the HIOs is that the enclosed lumen allows us to capture the cells to fully measure the extent of cell shedding, however in the intestine where there is flow these cells would be washed away and could help to reduce bacterial load in the intestine. This point is now made in lines 386-388 in the discussion.

Reviewer #2 (Evidence, reproducibility and clarity (Required)):

*Major concerns*

*1) The authors show that only ~5% of the neutrophils have migrated to the lumen, which is a barely noticeable increase compared to PBS treated organoids. Does this reflect that the mucosal layer of the organoids might not produce neutrophil chemoattractants and that neutrophil recruitment during Salmonella is a bystander effect from a different cell type?*

This number indicates that PMNs are ~5% of total cells in the PMN-HIO (including epithelial and mesenchymal cells) during *Salmonella* infection (not that only 5% of PMNs added migrated). Moreover, PMNs were added to a well containing multiple HIOs. We also show that HIOs do produce neutrophil chemoattractants during infection (S1 Fig).

*2) How quickly are neutrophils recruited to the HIOs? The authors show one time point of 8 hours. Related to the relatively low number of neutrophils seen in their HIOs, is this perhaps a result of the time point they chose? Will they see more neutrophils recruited if they go longer*?

It is likely that 5% of total cells in the PMN-HIO represents a significant recruitment of PMNs, and our data clearly indicate a marked effect on the infected epithelium. PMNs can cause substantial tissue damage, and their recruitment and activation is known to be tightly regulated. Due to the short-lived nature of human PMNs it would be difficult to extend this experiment to later timepoints. We have experimentally characterized PMN migration at 24h and by that time, most of the PMNs that we observe are non-viable, thus we focused our studies earlier.

*3) The authors show that PMNs did not kill STM in their organoids, but they do in pure culture. Is this simply because of the low levels of neutrophils present in their HIOs, which would result in lower concentrations of antimicrobials being produced in the HIO lumen? If the authors are able to get higher levels of neutrophils in their HIOs, would they see increased bacterial killing?*

Neutrophils have both inflammatory signaling and microbicidal functions. For example, Cho, et al (PLoS Pathogens 2012) find that neutrophil-derived IL-1 beta is sufficient to support abscess formation in the innate immune response to *Staphylococcus aureus* soft tissue infection. Similarly, a recent study showed that activation of neutrophils by keratinocyte defensins in a *S. aureus* skin infection led to neutrophil IL1 beta and CXCL2 release that amplified antibacterial defenses (Dong, et al Immunity 2022). Moreover, in the native environment of the gut with extensive microbiome colonization, direct neutrophil microbicidal activity might be less effective against infection than signaling. Recruitment of higher levels of neutrophils in vivo or in the HIO might exacerbate damage of the epithelial barrier. In the discussion, we speculate there may be proteins, like Elafin, that are upregulated during infection and inhibit some neutrophil functions as a trade-off to control host tissue damage. We reason that our data strongly support an inflammatory signaling role for neutrophils to promote innate immune responses of the intestinal epithelium.

*4) Related to the above point, if the authors treat their HIOs with known neutrophil chemoattractants, can they increase the number of neutrophils that migrate into their organoids?*

High levels of chemoattractants are already being produced in the HIO in response to infection (S1 Fig). The most effective number of neutrophils in the context of intestinal infection may not be the highest number, given that neutrophils can cause tissue damage. Since we see a marked phenotype with the neutrophils that are recruited, we propose that this PMN-HIO model reveals important inflammatory signaling roles for PMNs to promote intestinal epithelial immune function.

*5) The authors speculate that Salmonella may "employ specific mechanisms to overcome PMN effector functions in the HIO luminal environment". Are any such mechanisms known? If so, the authors could test this hypothesis by repeating these experiments with Salmonella mutants in which these mechanisms are ablated. In this case, they should see increased killing of Salmonella by PMNs in the HIO lumen.*

The focus of this study was to test how PMNs contribute to the host response against wildtype *Salmonella*. In the PMN-HIO model, we find that neutrophils direct a robust epithelial cell extrusion response, impacted intracellular bacterial numbers, and that *Salmonella* luminal colonization is not affected by PMNs. Thus, our data are pointing to an important inflammatory role for neutrophils in the infected intestine. Indeed, reliance on direct bactericidal mechanisms in the intestinal lumen which in vivo would be colonized with the microbiota might be a losing strategy for neutrophils, which would be hugely outnumbered.

*6) Furthermore there is no information of the activation status of the neutrophils. How does the surface expression of CD16 CD62L, CD66 and CD11b look between the migrated and non-migrated and between infected and uninfected controls? Did the neutrophils de-granulate? Are they CD63+ or is the high levels of NGAL and S100 proteins an effect of lysis? The authors should also be careful in claiming that there is NETosis as the image in the supplement look more like an artifact than actual NETs.*

Our new findings suggest that IL-1 production by PMNs is the biggest factor in driving the cell death phenotype. We have also added a figure with CD63 staining. We were able to visualize some localization of CD63 to the cell surface of PMNs, consistent with degranulation (S4 Fig)**.**

*7) Why does ASC translocate to the nucleus? Is the IL-1b cleavage mediated through Caspase-1 or Caspase-11? The neutrophils stained positive in the lumen appear to be intact, does this mean that pyroptosis does not occur, or does the IL-1b come from cells that did not migrate through the mucosal membrane? Staining for IL-1 and the different caspases might help resolve this question.*

ASC does not appear to be translocating to the nucleus. In Fig 3D the green signal (ASC) is primarily excluded from the DAPI-stained area. In this human model, Caspase-11 is not present, and inhibition of Caspase-1 is sufficient to block the cell shedding phenotype (Fig. 5A,B and Fig. 6B,C). We are unable to distinguish whether IL-1 is being produced by intact PMNs or PMNs that are undergoing pyroptosis. Unfortunately, there are not suitable antibodies for fixed immunofluorescence staining for cleaved Caspase-1, and as a secreted protein, IL-1 beta likely will not remain localized with the producer cell.

*8) The authors comment that there is substantial TUNEL staining in the mesenchyme independent of STM or PMNs, however, there is no explanation for why this happens. Does this have any downstream effects on the neutrophils that doesn't migrate towards the lumen?*

TUNEL positivity in the mesenchyme is likely an artifact of culturing and we have noted this in the text (line 169-172). The extent of TUNEL+ mesenchymal cells appears to be dependent on the batch of HIOs as not all HIOs exhibit this phenotype (for example Figs 4A and 6B). In contrast, the extent of TUNEL+ luminal cells is significantly dependent on the presence of PMNs and *Salmonella*.

*Minor comments*

*1) The authors should remove that MPO is neutrophil-specific, monocytes are known to have higher MPO expression than neutrophils*.

In this controlled co-culture system there are no monocytes, therefore we have modified our text to indicate that MPO is used as a neutrophil marker in the PMN-HIOs (line 161).

*2) If the authors performed flow cytometry as they say, they should provide the flow plots and the gating strategy they used in the supplement.*

Representative flow plots for the data presented in Fig 1A are now included in S2 Fig. The data shown in Figs. 1A and S2 Fig are not gated.

Reviewer #3 (Evidence, reproducibility and clarity (Required)):

Major Comments

*1.Overall the study is convincing and it is well-conducted. This reviewer found it surprising that the PMNs did not alter the total levels of STM in the HIOs as neutrophils are expected to control the infection. Can the authors elaborate on if the intraepithelial numbers are reduced, what happens to STM in the lumen? It would be convincing if the authors can extend the infection timeline to see if the neutrophils are capable of killing luminal STM.*

One of the limitations of the HIO model is the lack of flow in the lumen. It is likely that shed cells would be removed from the body following extrusion in vivo. In the HIOs, since the cells are trapped in the lumen, *Salmonella* could then reinvade and so this phenotype might be even stronger in a model that incorporates flow. We have added this point to the discussion (lines 387-390). Due to the short-lived nature of PMNs, it is difficult to extend the infection beyond 8h. While in vitro experiments with just neutrophils and STM as we and others have performed might set the expectation that neutrophils would alter luminal bacterial levels, there is little to no direct evidence that neutrophil bactericidal activity is critical in the context of the intestinal environment (vs. releasing ROS or inflammatory signals that may have complex indirect effects). Indeed, an advantage of the HIO model is that we are able to test the function of neutrophils in a multi-component system, but one that is still sufficiently simplified that we can do some mechanistic analysis.

*2-It would be powerful to conduct the caspase inhibition on neutrophils prior to HIO co-culturing to convincingly show that the effects of caspase inhibition effect neutrophils which in turn effect the epithelium disrupting the epithelial load of STM.*

We appreciated this suggestion. We pretreated the PMNs with a Caspase-1 inhibitor for 1h prior to co-culture with infected HIOs. We found that this was sufficient to block TUNEL cell accumulation in the lumen of infected PMN-HIOs. These results are now presented in Fig 6B,C.

*3- While other caspases are well-established to be involved in Salmonella-related cell death and epithelial shedding, why did the authors picked caspase 3 but not caspase 4/5 to show activation in Fig 2?*

We have now also tested the role of Caspase-4 on cell shedding using z-LEVD-fmk inhibitor. Consistent with prior published studies, we found that Caspase-4 inhibition reduced the accumulation of TUNEL-positive cells in the PMN-HIO lumen. These results are presented in Fig 5. There are no detectable levels of Caspase-5 in the HIOs (S9 Fig).

*Minor comments*

*Fig 1C It is not clear how the total bacterial burden was determined. Please include details such as the timepoint and sufficient details of the technique both in the results section and the legend*.

These details have been added in the figure legend (line 605-607). Briefly, HIOs were washed with PBS and homogenized in PBS at 8hpi. CFU/HIO were enumerated by serial dilution and plating on LB agar.

*2. Fig S2. Authors claim that the PMNs form NETs in the lumen, however, the marker used in the immunostaining is MPO. Although a NETting is seen in the images, MPO staining is not sufficient to claim these are NETs. Additional staining is required to show if the neutrophils in the lumen are intact or formed NETs*.

As noted in response to Reviewer #1, although we commented on NETs in our original manuscript, our conclusions do not rely on the presence or absence of NETs and our new data implicates PMN IL-1 as necessary and sufficient for the cell shedding phenotype. We have therefore removed the NET data and the reference to NETs. While NETs are potentially interesting in the context of intestinal infection, we understand the reviewer's concern about NETs and anticipate that a more quantitative characterization of NETs may be challenging given the structure and variability of the PMN-HIOs.
